# Intestinal probiotics restore the ecological fitness decline of *Bactrocera dorsalis* by irradiation

**DOI:** 10.1111/eva.12698

**Published:** 2018-10-09

**Authors:** Zhaohui Cai, Zhichao Yao, Yushan Li, Zhiyong Xi, Kostas Bourtzis, Zheng Zhao, Shuai Bai, Hongyu Zhang

**Affiliations:** ^1^ State Key Laboratory of Agricultural Microbiology Key Laboratory of Horticultural Plant Biology (MOE) China‐Australia Joint Research Centre for Horticultural and Urban Pests Institute of Urban and Horticultural Entomology College of Plant Science and Technology Huazhong Agricultural University Wuhan China; ^2^ Department of Microbiology and Molecular Genetics Michigan State University East Lansing Michigan; ^3^ Insect Pest Control Laboratory Joint FAO/IAEA Division of Nuclear Techniques in Food and Agriculture Vienna International Centre Vienna Austria

**Keywords:** dysbiosis, ecological fitness, gut microbiota, irradiation damage

## Abstract

The sterile insect technique (SIT) as an eco‐friendly and reliable strategy has been used to control populations of insect pests of agricultural, veterinary and human health importance. Successful applications of SIT rely on the high‐level ecological fitness of sterile males. A suitable and stable gut microbiome can contribute to the ecological fitness of insect by influencing their physiology, biochemistry and development processes. Here, we show that a shift in the gut bacterial composition and structure by sterilizing irradiation, characterized by a decrease in the major gut microbiota community Enterobacteriaceae, an expansion of the minor members (e.g., Bacillaceae) and a higher richness and diversity, is tightly linked to radiation‐induced ecological fitness (male mating competitiveness, flight capacity, survival rate and life span) decline in *Bactrocera dorsalis* (Hendel) sterile males. Function prediction of gut microbiota indicated that changes in microbiome taxonomy tend to drive microbiome functional shifts. A higher nutrient consumption of the flourishing minor gut microbiota may cause a decline in nutrients and energy metabolic activity of host and then result in the reduced ecological fitness of irradiated flies. Furthermore, we found that a gut bacterial strain *Klebsiella oxytoca* (BD177) can restore ecological fitness by improving food intake and increasing haemolymph sugar and amino acid levels of irradiated *B. dorsalis* flies. Our findings suggest that gut symbiont‐based probiotics can be used as agents for reversing radiation‐induced ecological fitness decrease.

## 1 INTRODUCTION

The sterile insect technique (SIT) has been used for decades to control insect pests of agricultural, veterinary and human health importance (Lees, Gilles, Hendrichs, Vreysen, & Bourtzis, 2015; Leftwich, Bolton, & Chapman, 2016). Compared with insecticide control strategies, SIT has several attractive features including species specificity and environment friendliness. Ionizing irradiation was used to sterilize insects, and these insects were subsequently handled, transported and released in the field, ideally only males (Lance & McInnis, 2005). However, irradiation may have negative side effects on male insects’ fitness, resulting in the reduced mating competitiveness of irradiated sterile male decline, despite the fact that irradiation can sterilize insects effectively. The decline of flight performance, female attraction and longevity of irradiated males in Tephritidae pests and mosquitoes has been documented (Helinski, Parker, & Knols, 2009; López‐Martínez & Hahn, 2014). These negative side effects may be due to radiation exposure that can impose various impacts on an organism, for example direct disruption of atomic structures, and indirect damage to nucleic acids, proteins and lipids by reactive chemical materials generated from the radiolysis of water (Azzam, Jay‐Gerin, & Pain, 2012). Subsequent and profound impacts of radiation on the organism can occur at both physiological and biochemical levels (Azzam et al., 2012). It is crucial that the released sterile males possess high mating competitiveness to successfully compete with wild males for mating with wild females (Lance & McInnis, 2005). Thus, the deleterious impact on the ecological fitness of the released insects has been one of the most considerable issues of SIT applications (Leftwich et al., 2016).

Symbiotic microorganisms of animals create a complex ecosystem that is strongly connected with the biology of the host and contributes to its health (Clemente, Ursell, Parfrey, & Knight, 2012). Gut symbiotic microorganisms affect insects in several ways such as aiding food digestion and detoxification (Ceja‐Navarro et al., 2015), providing essential nutrients (Storelli et al., 2011), regulating development and immunity (Kwong & Moran, 2016; Shin et al., 2011), protecting against infectious pathogens (Cirimotich et al., 2011), influencing the central nervous system function and behaviour such as mating preference (Ben Ami, Yuval, & Jurkevitch, 2010; Sharon et al., 2010) and aggregation behaviour (Wada‐Katsumata et al., 2015). Whereas gut symbiotic microbiota community is tightly linked to insect host ecological fitness, the mechanism of the ecological fitness damage induced by sterile dose ionizing radiation and the possible role of gut microbiota is largely not understood. In addition, whether manipulation of the gut microbiota is an alternative approach to improve the ecological quality of sterile male needs to be explored.

*Bactrocera dorsalis* is a destructive polyphagous and ecological invasive insect pest of tropical and subtropical species of fruit and vegetable crops (Wang, Jin, et al., 2014). While insecticides have been used to control this pest, insecticide resistance and environment pollution by chemical insecticides have been seriously limiting this type of control (Cheng et al., 2017). Thus, SIT can be an alternate strategy for management of the Oriental fruit fly. Previously, SIT has been used to control pest fruit fly species, including *Ceratitis capitata* (Hendrichs, Franz, & Rendon, 2009), *B. tryoni* (Collins, Weldon, Banos, & Taylor, 2009), *B. cucurbitae* (Mau, Jang, & Vargas, 2007) and *B. dorsalis* (Orankanok, Chinvinijkul, Thanaphum, Sitilob, & Enkerlin, 2007). SIT may have some limitations related to the ecological fitness of sterile male fruit flies, but manipulation of gut symbionts maybe an alternative strategy for improving the mass rearing efficiency of high‐quality sterile males (Ben Ami et al., 2010). Earlier, gut symbiotic bacteria community of Oriental fruit fly has been investigated; Enterobacteriaceae were the predominant families in different populations from laboratory reared and field collected samples (Wang, Jin, & Zhang, 2011; Wang, Jin, et al., 2014). The predominance of Enterobacteriaceae has also been observed in *B. minax* (Wang, Yao, Zheng, & Zhang, 2014), *C. capitata* (Behar, Yuval, & Jurkevitch, 2005) and several species of the genera *Anastrepha* (Ventura, Briones‐Roblero, Hernández, Rivera‐Orduña, & Zúñiga, 2018). In *C. capitata*, the core bacteria Enterobacteriaceae can help the host in nitrogen fixation and hydrolyse pectin substances in plants (Behar, Yuval, & Jurkevitch, 2008; Behar et al., 2005). Our previous studies showed that *B. dorsalis* adults were attracted by the metabolites of symbiotic Enterobacteriaceae (Shi, Wang, & Zhang, 2012). Recently, a study demonstrated that Enterobacteriaceae can degrade trichlorphon and increase the insecticide resistance of host (Cheng et al., 2017). The continuous presence and dominance of the Enterobacteriaceae family in the gut bacterial community of tephritidae fruit flies indicate an important functional role as symbionts in host fitness.

Here we aimed at verifying the role of gut microbiota in *B. dorsalis* sterile males and exploiting an applicable approach to improve the SIT implementation efficiency via targeting gut symbiosis bacteria. In this regard, we revealed a significant change in the diversity, abundance and function of the gut microbiota of *B. dorsalis* by irradiation. Meanwhile, the ecological fitness decline of sterile males was observed. Then, the gut bacteria reduced due to irradiation were isolated and cultivated. By oral re‐infection, we found that a gut bacteria strain *K. oxytoca* (BD177) can repair the host ecological fitness damage caused by irradiation. Finally, we found that *K. oxytoca* (BD177) re‐infection improved food intake, sugar and amino acid levels in the haemolymph of irradiated flies and the potential contribution of *K. oxytoca* (BD177) to repair radiation damage and improvement in the ecological fitness of sterile males.

## 2 METHODS

### 2.1 Flies rearing

*Bactrocera dorsalis* flies were collected from the Guangzhou, Guangdong province, China and have been reared for about 20 generations at the Institute of Urban and Horticultural Entomology of Huazhong Agricultural University. In each experiment, adult flies were maintained in cages at 27 ± 1°C under a 12‐hr light:12‐hr dark photoperiod and supplied with sucrose: yeast extract (3:1) and water, while larvae were raised in bananas (Wang, Jin, et al., 2014).

### 2.2 Irradiation procedures

About 3,000 pupae (2 days before emergence) were placed in a plastic box irradiated with 100 Gy dose (Shelly et al., 2010). Radiation treatment was conducted at a nearby commercial γ‐ray irradiation facility centre (Hubei Academy of Agricultural Sciences, Wuhan, Hubei Province). The radiation source was a Gamma Cell 220 Co^60^ irradiator, with an activity of 9.435 × 1,015 Bq (2.55 × 10^6 ^Ci) and a central dose rate of 8 Gy/min at the beginning of the tests. The mean dose rate of irradiation was 3.33 Gy/min. A metallic holder was used to place the plastic box (310 mm in length, 210 mm in width by 185 mm in height) containing pupae at the centre of the γ‐radiation field. Pupae of the control treatment were also subjected to the 30‐min bus ride to the Hubei Academy of Agricultural Sciences and back to the Institute of Urban and Horticultural Pests at Huazhong Agricultural University, but were not irradiated. Irradiated pupae and un‐irradiated (UN) pupae both were maintained until emergence.

### 2.3 Experimental design

**Figure 1:**
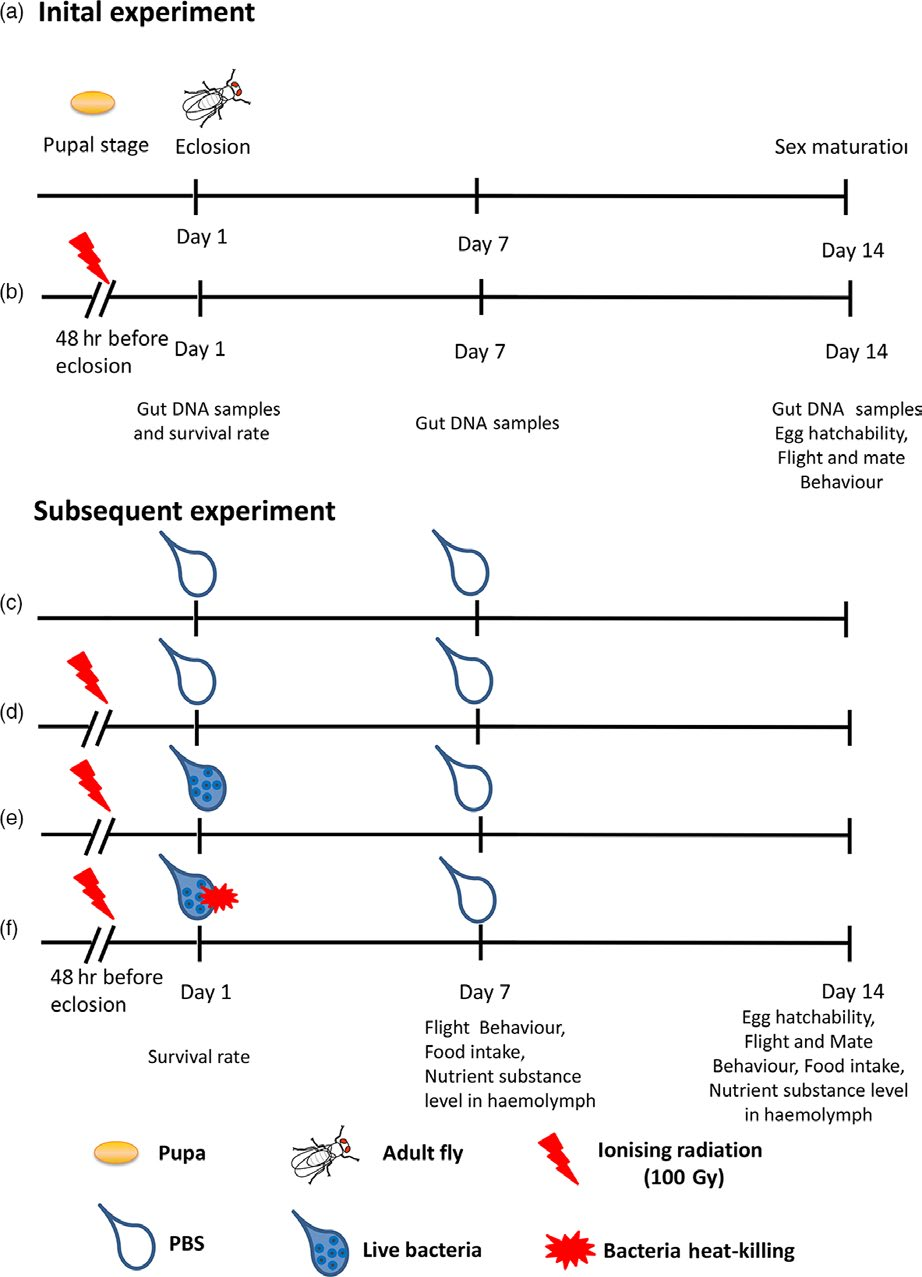
Study design timeline. Diagrammatic drawing representation of the initial and second experiments performed in this study. In initial experiment: (a) Un‐irradiated male flies + conventional diet and PBS. (b) Irradiated male flies + conventional diet and PBS. In second experiment: (c) Un‐irradiated males + conventional diet and PBS. (d) Irradiated males + conventional diet and germfree PBS. (e) Irradiated males + conventional diet and candidate‐bacteria enriched PBS. (f) Irradiated males + conventional diet and heat‐killing candidate‐bacteria enriched PBS. (c–f) After an experimental diet of 6 days, the dietary of all groups was replaced with the conventional diet and germfree PBS. Briefly, 15 flies per sample were used for total DNA extraction from the intestines. With a total of six samples at each time point, there were 90 flies used. So, at 1DPE (15guts *6 replicates=90guts), 7DPE (15*6 = 90), 14DPE (15*6 = 90) resulted in a total of 270 guts for irradiated flies, and 270 guts for un‐irradiated flies were used (540 guts in total). Simply, the 36 gut samples included 1DPE (6 samples), 7DPE (6 samples), 14DPE (6 samples) with a total of 18 samples for irradiated flies and in the same way total of 18 samples for un‐irradiated flies

The schematic experimental design is shown in Figure 1.

All assays were executed on the parental generation, and they were all siblings. The schematic experimental design is shown in Figure 1. We performed these experiments in two sections, for example initial and subsequent experiments as follows:

#### 2.3.1 Initial experiment

We investigated the impact of irradiation on the ecological fitness of *B. dorsalis* and compared the gut microbiota between the irradiated (IR) and UN group. Briefly, pupae (48 h before emergence) were irradiated with 100 Gy dose. Upon emergence (Figure 1a), un‐irradiated male flies were sorted out to raise and (Figure 1b) irradiated male flies were also separated to feed by their sex. Both groups were supplied with a conventional diet composed of sucrose: yeast extract (3:1) and germfree phosphate‐buffered saline (PBS). At one‐day post eclosion (DPE), six biological replicates of 15 guts were, respectively, collected from irradiated and un‐irradiated male group. Eclosion and survival rate were also tested. In the same way, at 7 DPE, each treatment sampled six biological replicates of 15 guts. At 14 DPE (sex maturation), six gut samples (15 guts each sample) for each treatment were collected. Behavioural parameters such as egg hatching rate, flight and mating were also detected. In total 36 gut samples, including both treatments, irradiated and un‐irradiated flies (6 biological replicates at each time point 1, 7, 14 DPE, in total 18 gut samples for each treatment) (Figure 1) were subjected to 16S *rRNA* gene amplicon sequencing. According to the gut microbiota analysis results, two candidate strains were obtained.

#### 2.3.2 Subsequent experiment

The function of the candidate strain (BD177), whether fitness parameters of sterile males recovered, was also verified. After eclosion, flies were housed in laboratory cages (30 × 30 × 30 cm), in groups of 150–200 individuals, for 6 days during which four different experimental diets were supplied to each group. (Figure 1c): Un‐irradiated males fed on the conventional diet and PBS. (Figure 1d): Irradiated males fed on the conventional diet and germfree PBS. (Figure 1e): irradiated males were fed on conventional diet and the candidate‐bacteria enriched PBS. (Figure 1f): Irradiated males were fed on conventional diet and heat‐killed (autoclaved) candidate‐bacteria enriched PBS. After an experimental diet of 6 days, the diet of all groups was replaced with the conventional diet and germfree PBS.

### 2.4 Isolation of bacterial DNA from the gut and high‐throughput sequencing

Flies were surface‐sterilized and extracted by dissection under sterile conditions using a microscope. The total bacterial DNA from the intestines (from crop to hindgut without Malpighian tubes) of 15 individuals per treatment was extracted for different experiments, using an E.Z.N.A.TM Soil DNA kit (Omega, USA) according to the manufacturer's instructions. Briefly, gut DNA was isolated by treating the homogenized gut in 1 ml of buffer SLX Mlus and 100 μl of buffer DS incubated at 70°C for 10 min. DNA was precipitated using a high‐salt/ethanol precipitation method and was washed extensively with 70% ethanol (Yao et al., 2016). A total of 36 gut samples were subjected to high‐throughput sequencing, including the un‐irradiated and irradiated male flies at three different time intervals, 1, 7 and 14 DPE. There were six biological replicates per treatment, and each biological replicate included 15 flies (Figure 1a,b). To assess the microbial diversity of the flies, bacterial 16S ribosomal DNA amplicons were prepared by amplification of the V4 hypervariable region of the 16S rDNA with universal primers (U515F/806R) flanked by Illumina standard adapter sequence (Caporaso et al., 2011; Walters et al., 2011). The PCR products were checked using 2% agarose gel electrophoresis, purified using the AxyPrep DNA gel extraction kit and quantified using Qubit 3.0 Fluorometer (Life Technologies, Grand Island, NY). The purified amplicons were sequenced on an Illumina MiSeq PE250 sequencer at the BGI (Beijing Genomics Institute, Shenzhen, China).

### 2.5 Bioinformatics and amplicon analysis

The FLASH was used for extending the length of short reads by overlapping paired‐end Illumina MiSeq DNA reads for genome assemblies (Magoč & Salzberg 2011). Usearch (v7.0.1090) was used to clean contigs and remove those with E > 0.5 (Edgar, 2010). Contigs were clustered to 97% identity against DNA sequences in the Greengenes database (version 2013.5) (DeSantis et al., 2006), using the QIIME, version 1.8 (Caporaso et al., 2010). To focus the analysis on bacterial taxa, >97% of operational taxonomic units were picked using a closed‐reference operational taxonomic unit picking protocol against Ribosomal Data Project II database (Cole et al., 2005). Richness and diversity indices (Observed species, Chao1, abundance‐based coverage estimator (ACE), (Shannon and Inverse Simpson) and dissimilarity matrices (Bray–Curtis and weighted UniFrac) were estimated using the mothur software package (Schloss et al., 2009). Measurements of beta‐diversity were facilitated and visualized using principal coordinate analysis, PCoA, as implemented in QIIME (Caporaso et al., 2010). LefSe (Segata et al., 2011) was used to identify genera of most consistently different between sample types. To explore the metabolic activity of the bacterial communities found on the gut contents of different treatment groups, a bioinformatics tool PICRUSt (Langille et al., 2013) was used to generate the KEGG (Kyoto encyclopaedia of genes and genomes) pathway.

### 2.6 Real‐time quantification of the microbiota

The 36 DNA samples (six replications per treatment) subjected to high‐throughput sequencing were also detected for the absolute quantification of gut microbiota. β‐Actin and 16S *rRNA* gene primers specific to total bacteria and Enterobacteriaceae were used to quantify commensal microbiota to confirm the information on relative abundance from Illumina sequencing. Primer pairs used in quantitative PCR analysis were as follows: β‐actin (F 5′‐TCGATCATGAAGTGCGATGT‐3′ and R 5′‐ATCAGCAATACCGGGGTACA‐3′) (Yao et al., 2016), total bacteria (Eub338F: 5′‐ACTCCTACGGGAGGCAGCAG‐3′, Eub518R: 5′‐ATTACCGCGGCTGCTGG‐3′) (Guo, Xia, Tang, & Wang, 2008), Enterobacteriaceae (En‐lsu‐3F: 5′‐TGCCGTAACTTCGGGAGAAG GCA‐3′, En‐lsu‐3_R: 5′‐TCAAGGACCAGTGTTCAGTGTC‐3′) (Matsuda et al., 2009). Real‐time quantitative PCR was performed as previously described (Yao et al., 2016). Briefly, triplicate 20 μl reactions were used with 10 μl of 2 × Power SYBR Green Mix (Bio‐Rad), 0.4 μl of each 10 mM primer and 5 ng of template DNA on a Bio‐Rad 2000 real‐time PCR system. The amplification program consisted of (a) preincubation at 50°for 2 min, and 95°C for 10 min; (b) 45 cycles of denaturation at 95°C for 15 s and annealing at the appropriate temperature 60°C for 1 min; and (c) one cycle at 95°C for 15 s, 53°C for 15 s and 95°C for 15 s.

### 2.7 Isolation and identification of culturable bacteria

At 1 DPE, the quantity and diversity of culture‐dependent bacteria communities from un‐irradiated and irradiated male fruit fly group were detected. Briefly, different serial dilutions, for example 10^−5^, 10^−6^ and 10^−7^, of the homogenized gut extracts of two different groups were inoculated in triplicate, on Luria‐Bertani (LB) solid media under aerobic conditions with the spread‐plate method. After incubation at 30°C for 24 hr, individual, morphologically distinct colonies were made to count for colony forming units (CFU). Afterwards, these individual colonies were inoculated into the corresponding liquid media and shaken at 200 rpm and 30°C overnight to produce biomass for DNA extraction, and to preserve in 60% sterile glycerol stocks that were placed at −80°C for storage. The bacterial DNA was extracted with the HiPure Bacterial DNA Kit (Magen) following the protocol for Gram‐positive bacteria and used for the amplification of 16S *rRNA* genes using the primers 27F (5′‐GTTTGATCCTGGCTCAG‐3′) and 1492R (5′‐GGTTACCTTGTTACGA CTT‐3′) (Ceja‐Navarro et al., 2015). Subsequently, the ~1.4 kb PCR products were purified using a PCR purification kit (Axygen) and were subjected to bidirectional Sanger sequencing. The phylogenetic relationships analyses of 486 cultivable gut bacteria strains were performed. The maximum‐likelihood built with IQ‐TREE (Nguyen, Schmidt, von Haeseler, & Minh, 2014) is based on 16S *rRNA* gene aligned by indicating the relationship between 486 gut bacteria strains with 63 reference bacteria from NCBI database. Briefly, alignments of 486 16S *rRNA* gene sequences were generated using MUSCLE (Edgar, 2004). Alignments were trimmed for poorly aligned regions and gaps using the BMGE tool (Criscuolo and Gribaldo, 2010). Phylogenetic analyses were then carried out using IQ‐TREE, which implements an ultra‐rapid bootstraps and ModelFinder, a new model‐selection method that greatly improves the accuracy of phylogenetic estimates. Having found the optimal model TIM3+F+R4 according to the Bayesian information criterion (BIC), we then inferred the most likely tree for it using IQ‐TREE, with bootstrap scores inferred using the ultrafast bootstrap method. The phylogenetic tree was visualized by FigTree.

### 2.8 Fitness test

All assays were performed on the F0 generation.

#### 2.8.1 Eclosion rate

Emergence tests were conducted as previously described (Steinitz, Sadeh, Kliot, & Harari, 2015) with modifications as follows. A total of 100 pupae non‐irradiated in a plastic bowl (φ13.5 cm) with vermiculite were placed in a screened cage (30 × 30 × 30 cm), and the emergence of flies was recorded for 5 days. Five replicate cages were used for each group, Flies were separated into two categories: uneclosed (UE) and partially eclosed (PE: fly not fully separated from the puparium). Percentage of emergence was determined as (100−[UE + PE]).

#### 2.8.2 Feeding assay

The feeding assay was performed as previously described (Shin et al., 2011) with some modifications as follows: Under starvation for 24 hr, adult males were exposed to liquid artificial diet (sucrose:yeast extract:water (3:1:40) w/w/w) containing 0.16% (w/v) FD&C Blue #1 dye for 45 min. Four flies per group were homogenized in 1,000 μl of PBS, and the homogenate was centrifuged at 12,000 *g* for 5 min, 1.2 ml PBS was added to 0.3 ml of the supernatant and centrifuged again for 5 min. Following centrifugation, the supernatant was loaded into a 96‐well crystal plate and the relative amount of dye ingested was quantified as the optical density (OD) of the sample at 633 nm (Bio‐Rad Company.), subtracting the background OD of a corresponding group of flies being fed without dye mixture. Aliquots of dye‐containing food were also measured using a similar method to estimate the mass of consumed food.

#### 2.8.3 Longevity

To test the effect of irradiation on longevity, 1‐day‐old irradiated and control fully eclosed males (*n* = 70~76 individuals from each treatment) were placed in a laboratory cage containing a diet mixture of sucrose and yeast powder at ratio 3:1+ water. The cages were inspected every day, and the number of dead flies was noted.

#### 2.8.4 Mating tests

Virgin female flies were separated from males <24 hr after eclosion and provided with water and a diet of sucrose and yeast powder (3:1) until sexual maturity. Groups of 12‐ to 14‐day‐old male flies from different treatments were each marked with a different Day‐Glo dye, which does not affect survival (Serghiou, 1977). Mating trials were started by placing 20 irradiated males and 20 non‐irradiated males into cages (30 × 30 × 30 cm). This was followed by introducing 20 virgin females 5~10 min later. Flies that were found dead, incapable of flight, or noticeably damaged in any way at the time of release were replaced. Tests began at 18:00 and lasted for 4 hr during which mating pairs were collected and scored as irradiated or non‐irradiated males and placed in one of two fresh rearing cages (irradiated or non‐irradiated). Next, 150 eggs were collected from each group, cultured on wet filter paper and maintained at 28 ± 1°C for detecting hatchability (Zheng, Liu, Zheng, Xiao, & Zhang, 2015). This trial was replicated thrice. For a given replicate to be included in the analysis, at least 20% of the females needed to be collected in mating pairs.

#### 2.8.5 Measurement of flight capacity

We used a computer‐monitored flight mill (Jiaduo Industry & Trade co., LTD, Hebi, China), custom‐made for the monitoring of insect flight ability, similar to the equipment described in previous research (Attisano, Murphy, Vickers, & Moore, 2015). Flight mills were linked to a recorder, which was connected to a computer and placed in a room where temperature, RH and light intensity could be adjusted. Flies were immobilized by placing them on ice. Then same size adult males were put on glass slides and mounted, via the pronotum, on 13‐cm‐long segments of steel wire (0.4 mm in diameter) with a droplet of Glue (Loctite 401, Loctite Corp, Rocky Hill, CT) within 7th‐ and 14th‐day post emergence. The steel cantilever was placed between two miniature magnets on the flight mill immediately thereafter, to ensure horizontal placement of fly for the sake of smooth flight. Data recorded by the software included the time of flight initiation, each mill revolution and the number of revolutions that occurred in consecutive 5‐s intervals. Flights 5 min apart were considered to be separate flights. Twelve males were tested at one time. Each test was conducted for 4 hr. The total number of flight mill revolutions was recorded and flight distance, speed, duration and the maximum speed of each flight for each one were computed using a custom‐made software package (Chen et al., 2015). During the process, the temperature was maintained at 25°C, 60% RH and a light intensity of 1.2205 klx.

#### 2.8.6 Sugar, triacylglyceride and amino acid measurement

Haemolymph was collected from adult male flies using a centrifugation procedure previously described (Dong, Li, Li, Jia, & Zhang, 2017) with modifications. In brief, after immobilization on the ice, an incision was made across the pronotum wall with a microneedle which was prepared with a puller at 60.8°C (PC‐10, Narishige, Tokyo, Japan). Eight killed flies were then placed in a 0.5‐ml microcentrifuge tube that was punctured with thumbtack at the bottom. The 0.5‐ml tube was then inserted into a 1.5‐ml microcentrifuge tube. After centrifugation at 514 g for 10 min, haemolymph was collected at the bottom of 1.5 ml tube. The collected haemolymph was used immediately or stored at −80°C. Sugar and triacylglyceride measurements were performed as previously described (Shin et al., 2011). Amino acid measurements were performed using Total Amino Acid assay kit (Jiancheng, Nanjing, China) according to the manufacturer's instructions.

### 2.9 Statistical analysis

All of the results from two independent groups were analysed using Student's *t* test or Mann–Whitney *U* test using SPSS18 (IBM Corporation, Armonk, NY, USA). Survival statistics were processed using log‐rank analysis and the Gehan–Breslow–Wilcoxon Test. Graphs were made by GraphPad Prism 6.0 (GraphPad Software, La Jolla, CA, USA) or Microsoft Excel (Microsoft, Redmond, WA, USA). If not otherwise stated, statistical significance was indicated as follows: ***, *p* < 0.001;**, *p* < 0.01; *, *p* < 0.05; and not significant (n.s).

### 2.10 Data accessibility

The entire 16S *rRNA* gene Illumina sequence dataset can be retrieved from the National Center for Biotechnology Information Sequence Read Archive (accession no. PRJNA413124).

## 3 RESULTS

### 3.1 The relationship between intestinal bacterial communities and ecological fitness damage of *Bactrocera dorsalis*

#### 3.1.1 Fly fitness is impaired by ionizing radiation

**Figure 2:**
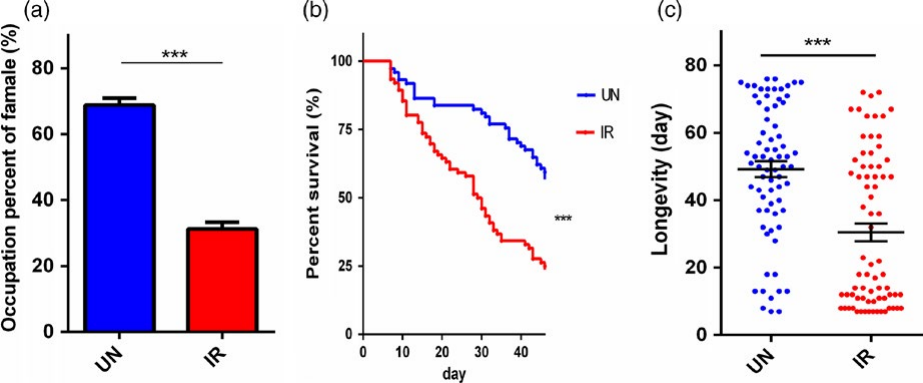
Influence of γ‐irradiation on male flies’ fitness. (a) Mating competition test. Irradiated male flies competed with un‐irradiated male flies mating for virgin females flies (*n* = 6). (b) Survival rate. Survival curves of *B. dorsalis* by log‐rank analysis (individual = 75). (c) The scatter diagram of the adult longevity. IR: irradiated male flies; UN: un‐irradiated male flies. Data were analysed using Student's test (a and c) and Gehan–Breslow–Wilcoxon test (b). The error bars indicate standard error (*SE*). (*p < 0.05, **p < 0.01, ***p < 0.001)

Irradiated male flies were significantly less fit as measured by mating competitiveness, flight capacity, survival rate and life span. Irradiated male flies exhibited shorter flight duration (*p* < 0.01), shorter cumulative flight distance (*p* < 0.05), slower average flight speed (*p* < 0.005) and slower top flight speed (*p* < 0.001) than non‐irradiated (UN) male flies at sexual maturation, that is, 14 DPE (Table 1). At 14 DPE, only 31% of IR males succeeded in mating, which is significantly lower (*p* < 0.0001) than the 69% success rate for UN males (Figure 2a). Ionizing radiation reduced the median survival of the IR flies to 30 days as compared to a median survival of 51 days of the control group, which represents an approximately 46% decrease (Figure 2b). In addition, there was a significant decrease (*p* < 0.0001) of the mean longevity in the IR group as compared to the control group (Figure 2c). As expected from and desired for SIT applications, the irradiation induced a significant decline in male fertility: the egg hatching rate of normal females that mated with IR males was 16%, which was significantly lower (*p* < 0.0001) than the egg hatching rate of 84% observed in normal females mated with UN males (Supporting Information Figure S1). These experiments showed that ionizing radiation severely decreases the ecological fitness of male flies.

**Table 1:** Flight parameters of *B. dorsalis* adult males at 14 DPE

	Mean accumulative flight duration (min) (mean ± *SE*)	Mean accumulative flight distance (km) (mean ± *SE*)	Mean flight speed (m/s) (mean ± *SE*)	Mean fastest flight speed (m/s) (mean ± *SE*)
UN	64.51 ± 9.93	1.99 ± 0.31	1.86 ± 0.12	3.40 ± 0.16
IR	34.25 ± 7.67**	0.82 ± 0.18*	1.35 ± 0.07***	2.69 ± 0.17**

*Notes*

Tested at 26°C, no wind and 60% RH for tethered‐flight 4 hr, *n* > 15. Data were analysed using Student's test. The error bars indicate standard error (*SE*). (*p < 0.05, **p < 0.01, ***p < 0.001).

IR: irradiated flies feeding on conventional diet only; UN: un‐irradiated male flies feeding on conventional diet only.

#### 3.1.2 Ionizing radiation increase microbial community diversity and richness

**Figure 3:**
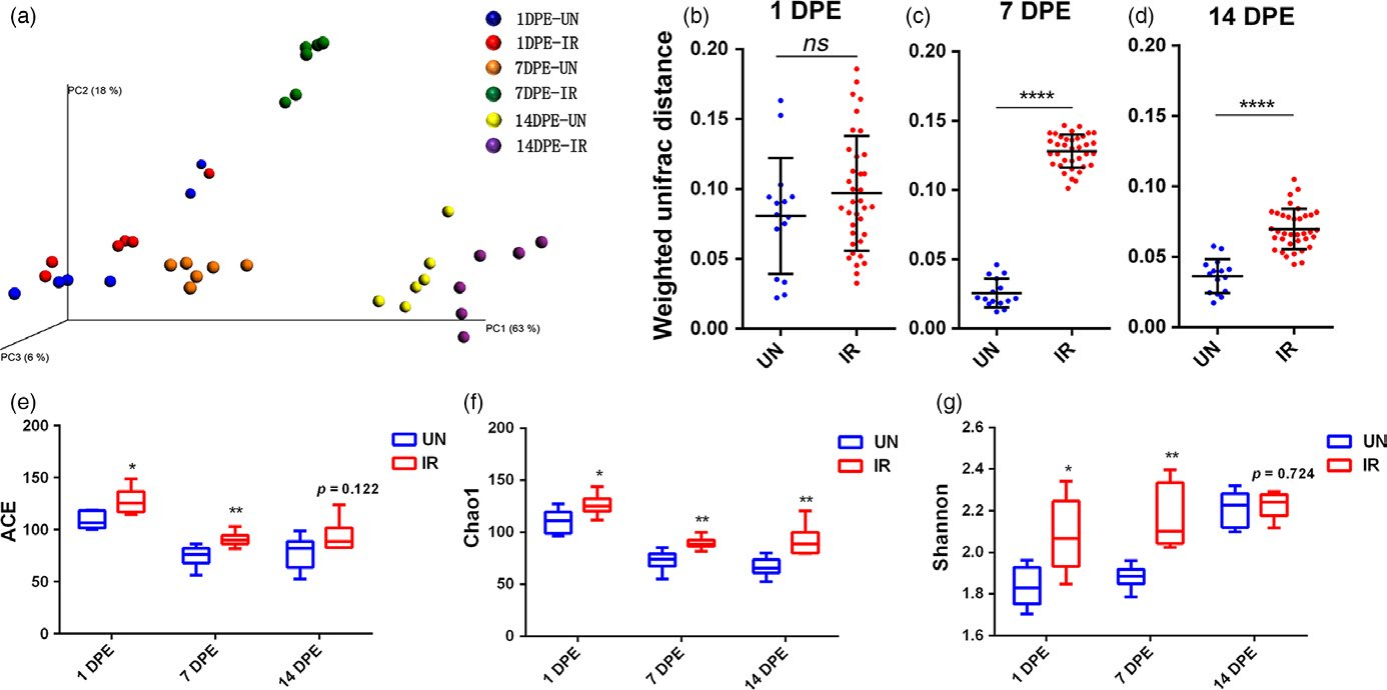
Diversity metrics of microbiota community explained by irradiation and male flies age. (a) Principal coordinate analysis based on weighted UniFrac metrics. (b~d) Scatterplots of distances between irradiated microbiota themselves and between irradiated and un‐irradiated microbiota at 1 DPE (b, *n* = 15~32), 7 DPE (c, *n* = 15~32) and 14 DPE (d, *n* = 15~32), respectively. The median is plotted as a horizontal line. Statistical comparison was based on nonparametric Mann–Whitney *U* test (****p < 0.0001). (e~f) ACE (e, *n* = 6), Chao1 (f, *n* = 6), and Shannon (g, *n* = 6) indices of gut bacterial communities from different treatment at three time points. Statistical comparison was based on Student's *t* test. The error bars indicate standard error (*SE*). (*p < 0.05, **p < 0.01). IR: irradiated male fly; UN: un‐irradiated male fly

To investigate the role of gut microbiota in ecological fitness decline of sterile males by irradiation, we examined the gut microbial composition and structure of the male flies by high‐throughput sequencing of the 16S *rRNA* gene at each of 1, 7 and 14 DPE with and without ionizing irradiation. Operational taxonomic unit (OUT) cluster analysis revealed profound shifts in the bacterial community composition along with irradiation treatments at different developmental periods of adults, as shown in the principal coordinate analysis based on the weighted UniFrac metrics, which measures phylogenetic dissimilarities between microbial communities. A three‐dimensional PCoA analysis showed a separation of IR and UN samples along the first three axes that explained 63%, 18% and 6% of data variation, respectively (Figure 3a). Notably, the average UniFrac distance between IR and UN samples was significantly larger than the intra‐group UniFrac of UN samples at 7 and 14 DPE (Figure 3c,d), However, there was no significant difference at 1 DPE (Figure 3b), indicating significantly greater phylogenetic difference between IR and UN samples than within UN themselves along with time.

The influence of irradiation on the abundance‐based coverage estimator (ACE), richness (Chao1) and community evenness (Shannon H) of *B. dorsalis* gut microbiota were also investigated. The results showed that irradiation induced a significant increase in the fly gut microbial diversity at 1 DPE as shown by the ACE, Chao1, Shannon indexes (Figure 3e~f). At 7 DPE, the ACE, Chao1 and Shannon indexes were 15%, 22% and 60% significantly higher, respectively, in the irradiated flies compared with the un‐irradiated flies (Figure 3f). Consistently, there was also a significant difference (*p* < 0.05) in the Chao1 index between irradiated and control flies, at 7 DPE. Altogether, these results suggested that irradiation significantly increased the microbial diversity and richness, which could not be reverted by the host itself.

#### 3.1.3 Ionizing radiation alters the composition and structure of intestinal microbiota community

**Figure 4:**
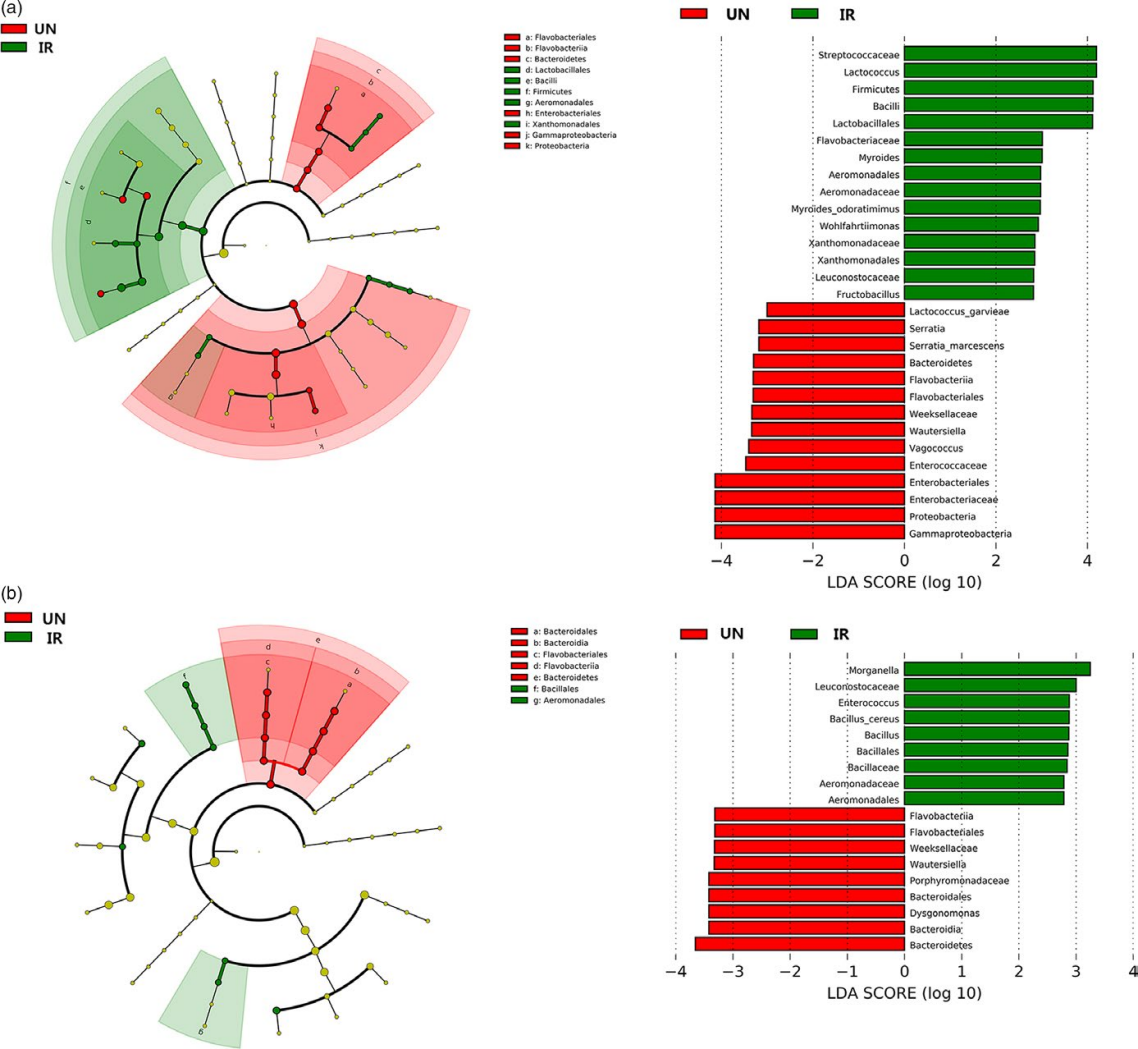
Irradiation induced changes in composition and structure of intestinal microbiota community. (a, b) Bacterial phylotypes of which abundances were changed in irradiated fly compared to un‐irradiated fly at 7 DPE (a) and 14 DPE (b) identified by LEfSe. The taxa listed in green most consistently describe irradiated samples. In contrast, those listed in red most consistently describe un‐irradiated samples. (a) At 7 DPE, cladogram (left) derived from LEfSe analysis of 16S *rRNA* gene sequences from irradiated samples and un‐irradiated samples. Green shaded areas indicate bacterial taxa that more consistently describe the irradiated samples; whereas red‐shaded areas indicate those that more consistently describe the un‐irradiated samples. Linear discriminant analysis (LDA) score (right) on the *x*‐axis represents log changes in relative bacterial phylotypes representation in un‐irradiated compared to irradiated flies at the upper right of the figure. (b) At 14 DPE, cladogram (left) derived from LEfSe analysis of 16S *rRNA* gene sequences from irradiated samples and un‐irradiated samples. Green‐shaded areas indicate bacterial taxa that more consistently describe the irradiated samples; whereas red‐shaded areas indicate those that more consistently describe the un‐irradiated samples. Linear discriminant analysis (LDA) score on the *x*‐axis represents log changes in relative bacterial phylotypes representation in un‐irradiated compared to irradiated flies at the bottom right of the figure. IR: irradiated sample; UN: un‐irradiated sample

Ionizing radiation also altered the composition and structure of the intestinal microbiota community. To identify key phylotypes responsible for the differences observed at 7 and 14 DPE and identified 29 phylotypes as high‐dimensional biomarkers at 7 DPE (Figure 4a), the linear discriminant analysis effect size (LEfSe) method was employed. The IR samples were characterized by a preponderance of Lactococcus, Streptococcaceae, Lactobacillales, Bacilli, Firmicutes; Myroides odoratimimus, Myroides, Flavobacteriaceae, Xanthomonadaceae, Xanthomonadales; Aeromonadaceae, Aeromonadales; Wohlfahrtiimonas and Fructobacillus, Leuconostocaceae. However, *Serratia marcescens*, Serratia, Enterobacteriaceae, Enterobacteriales, Gammaproteobacteria Proteobacteria; Vagococcus, Enterococcaceae; Wautersiella and Weeksellaceae, Flavobacteriales, Flavobacteriia; *Lactococcus garvieae*, Bacteroidetes were more consistently present in UN samples as compared to the IR samples (Figure 4a). At 14 DPE, *Bacillus cereus*, Bacillus, Bacillaceae, Bacillales, Morganella, Enterococcus, Leuconostocaceae, Aeromonadaceae, Aeromonadales were more constantly present in IR samples than that in the UN samples, which were characterized by a preponderance of Weeksellaceae, Wautersiella, Dysgonomonas, Bacteroidetes (Figure 4b). Quantitative histogram of differential features was generated from family level per cent relative abundance data showing means with standard deviation (Supporting Information Figure S2). Furthermore, a load of total bacteria, as well as Enterobacteriaceae in the gut of the samples, was quantified by real‐time PCR (Supporting Information Figure S3). The load of total bacteria and Enterobacteriaceae in the gut of IR male flies decreased by ~40% and 54%, respectively, as compared to that in control group at 1 DPE (Supporting Information Figure S3a,b). No significant differences were observed in loads of total bacteria at either 7 or 14 DPE (Supporting Information Figure S3c,e) but the loads of Enterobacteriaceae were consistently lower in the IR group, that is ~52% and ~51% lower than the control group at either 7 and 14 DPE, respectively (Supporting Information Figure S3d,f).

#### 3.1.4 Gut microflora function of male flies is perturbed by ionizing radiation

To identify shifts in the functional capacity of the community associated with irradiation, the PICRUSt was employed to predict the overall function of the *B. dorsalis* gut microbiome at 7 and 14 DPE. It was determined that the irradiation group exhibited a different relative abundance of the level two gene functions (Supporting Information Figure S4a,b). At 7 DPE, there were 14 gene categories each with a significant relative abundance (*p* < 0.05) that differed between the two groups of samples. We also noticed that the relative abundance of primary nutrient‐ and detoxication‐related metabolism genes such as carbohydrate, lipid, amino acid and xenobiotics biodegradation metabolism was significantly higher (*p* < 0.05) in IR flies (Supporting Information Figure S4a). At 14 DPE, 10 gene categories were found with a significantly different relative abundance level, indicating that the gut microflora of IR flies had lower carbohydrate, lipid, amino acid, cofactor and vitamin metabolism than that of UN flies (Supporting Information Figure S4b).

**Figure 5:**
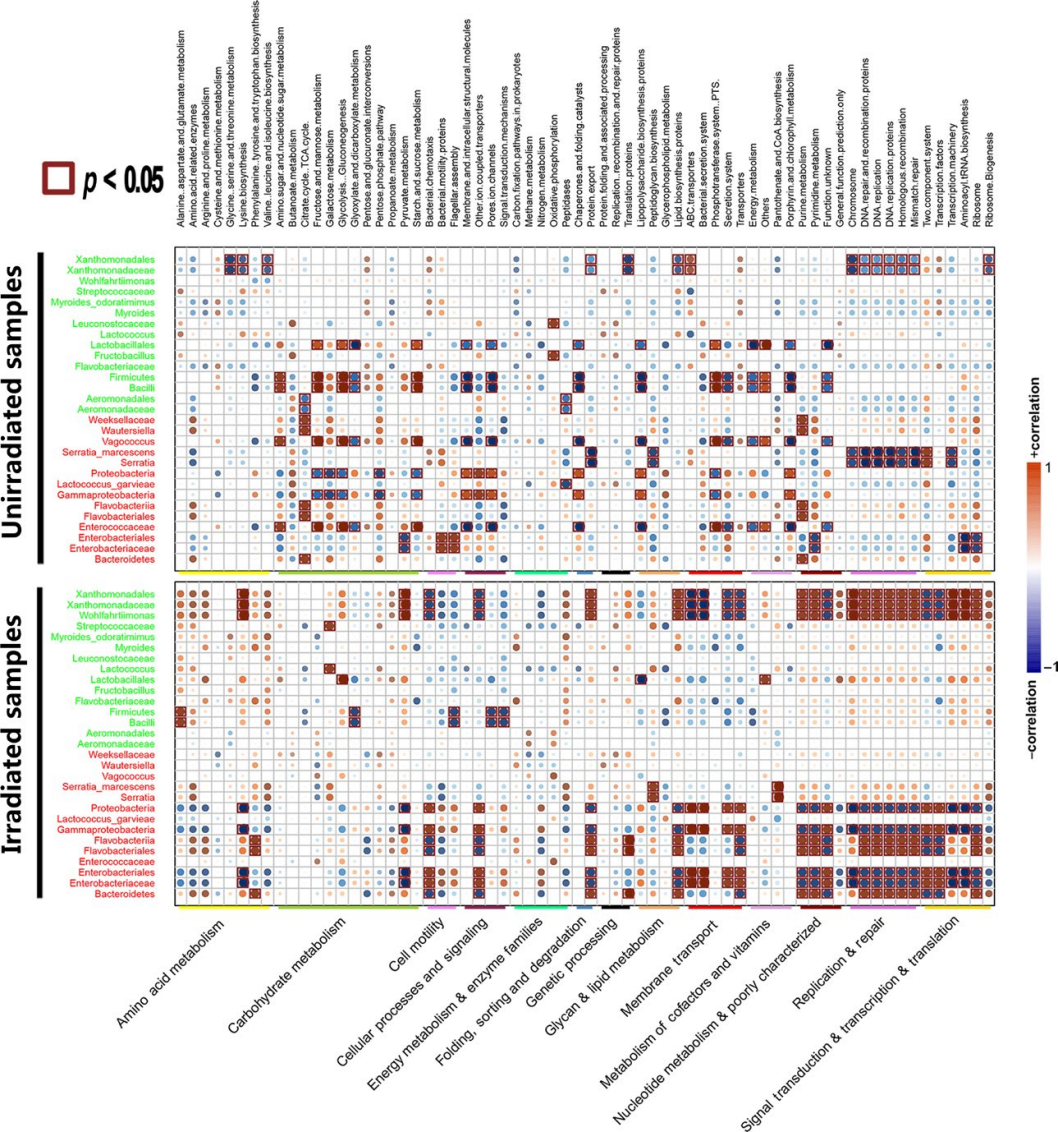
Bacterial metabolic and other pathway differences in the gut samples of UN versus IR male fly at 7DPE. Correlation analyses between the PICRUSt‐generated functional profile and LEfSe analysis‐generated bacteria groups that differed were performed for various KEGG metabolic and other pathways in UN and IR male fly at the individual time points of 7 DPE. Metabolic pathway designations are delineated at the bottom of the figure. Shading intensity and size of the circles indicates the Kendall rank correlation coefficient between matrices. Orange/red indicates a positive correlation; whereas blue designates a negative correlation. Red squares surrounding the circles are indicative of a *p* value ≤0.05. As in Figure 4, taxa listed in green were indicative of characteristic microflora in IR samples. In contrast, those listed in red were indicative of characteristic microflora in UN samples. IR: irradiated sample; UN: un‐irradiated sample

Next, we used PICRUSt to investigate key phylotypes likely causing the gut microbiome function changes by correlating the bacteria groups identified in the LEfSe analyses at 7 DPE (Figure 4a) with biological pathways (e.g., KEGG). At 7 DPE, the increased relative abundance of the minor members of the gut community, including Xanthomonadales, Xanthomonadaceae, Wohlfahrtiimonas, *Lactococcus*, Streptococcaceae, Lactobacillales, Bacilli and Firmicutes (Figure 4a), was positively correlated with an increase in the abundance of Amino Acid Metabolism, Carbohydrate Metabolism, Glycan & Lipid Metabolism, Metabolism of Cofactors and Vitamins, Replication & Repair categories in IR male flies (*p* < 0.05). In contrast, Cell Motility, Cellular Processes and Signaling and Membrane Transport categories were negatively correlated with the increased abundance of these bacteria groups in irradiated male flies compared with the UN male flies (*p* < 0.05). However, in the UN male flies, the elevated relative abundance of the major members of the gut community, especially Enterobacteriaceae (Figure 5), was only positively correlated with an increase in the abundance of Cell Motility. In contrast, Carbohydrate Metabolism, Nucleotide Metabolism and Signal Transduction & Transcription & Translation were negatively correlated with the increased abundance of these the major members of the gut community.

#### 3.1.5 Influence of γ‐irradiation on cultivable bacteria community in male flies

**Figure 6:**
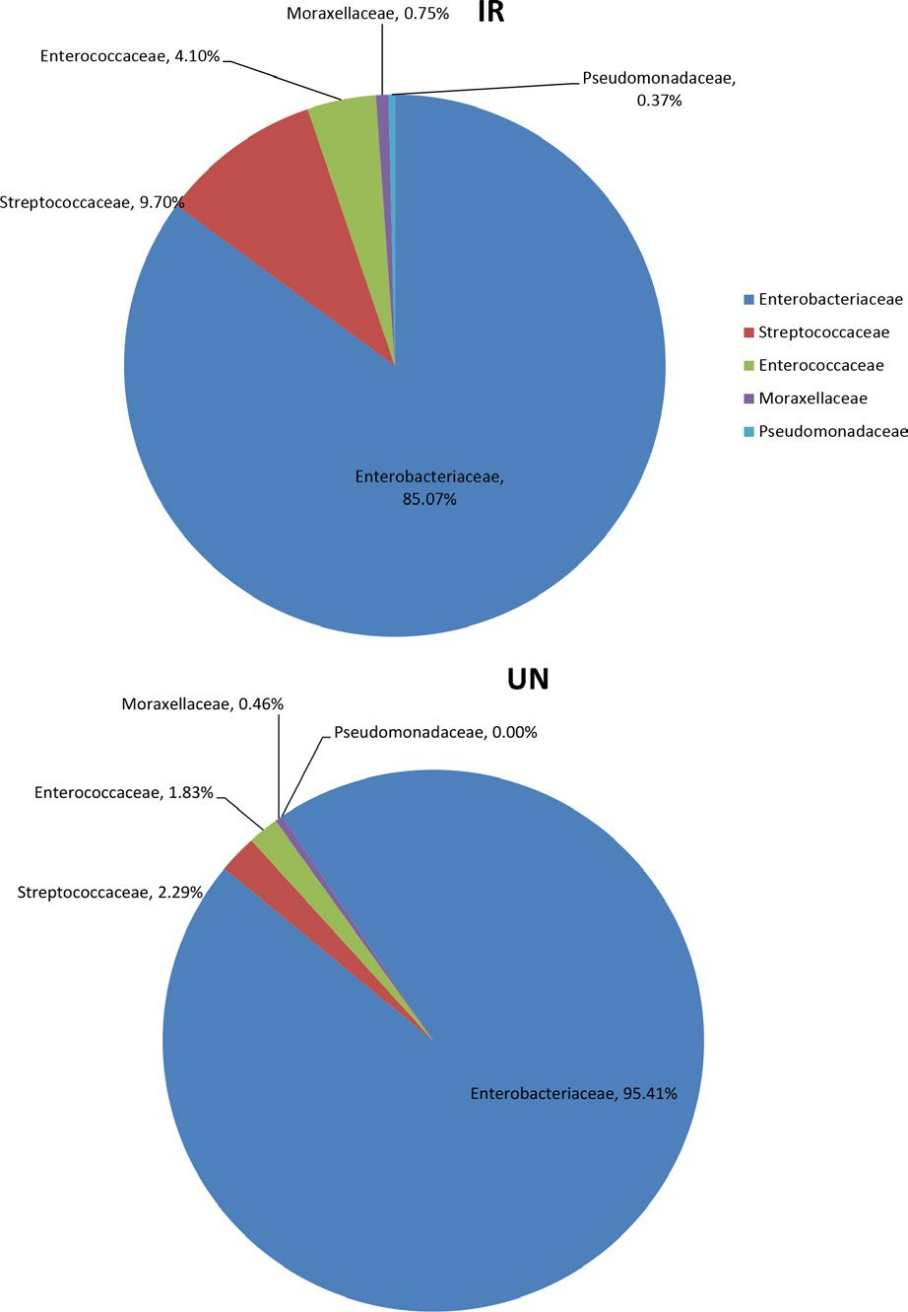
Irradiation‐induced the changes in culture‐dependent gut bacteria community. Pie charts represent the relative abundance of culture‐dependent gut bacteria community in family level. IR: irradiated samples; UN: un‐irradiated samples

The above results strongly suggest that the host ecological fitness damaged by irradiation may be interlinked with dysbiosis of intestinal microbial community. To acquire key gut bacterial strains influencing host ecological fitness, that restore damaged ecological fitness by manipulating the intestinal microbial community, we isolated and cultured bacteria using the Luria‐Bertani (LB) solid media, collected from the digestive tracts of UN and IR insect samples of 1 DPE as inoculum. We found that the cultivable gut bacterial load decreased significantly (by approximately 12~450‐fold) in IR samples compared to UN samples at three time points (Supporting Information Figure S5). A total of 486 isolates including 268 from IR and 218 from UN were obtained from the families Enterobacteriaceae, Enterococcaceae, Streptococcaceae, Moraxellaceae and Pseudomonadaceae (Figure 6). Consistent with Illumina sequencing results, we found that the relative abundance of Enterobacteriaceae was lower in IR flies (81.82%) than that in UN flies (95.41%), but the relative abundance of Enterococcaceae and Streptococcaceae in IR samples was 2~4 times higher than that in the control. Interestingly, Pseudomonadaceae were only detected in the IR flies (Figure 6). A maximum‐likelihood built with IQ‐TREE based on 16S *rRNA* gene indicated the relationship between 486 gut bacteria strains with 63 reference bacteria from NCBI database. It indicated the similarity between these bacteria with reference strain (Supporting Information Figure S6). Overall, 17 representative bacterial strains were identified and the relative abundance of these bacteria strains in IR group and UN group was listed in Table 2. The relative abundance of three major strains (>2% representation), *Citrobacter koseri* (BD195), *Klebsiella oxytoca* (BD177) and *Enterobacter soli* BD400 in IR gut decreased by 81.82%, 54.17% and 33.75%, respectively, compared with the UN samples (Table 2).

**Table 2:** The relative abundance of representative 17 bacterial strains in irradiated group and un‐irradiated group

Representative bacterial strain	IR	UN	Percentage change (IR‐UN)/UN
*Enterobacter soli* BD473	53 (19.78%)	80 (36.70%)	−33.75%
*Enterobacter tabaci* BD138	39 (14.55%)	35 (16.06%)	11.43%
*Klebsiella oxytoca* BD177	11 (4.10%)	24 (11.01%)	−54.17%
*Enterobacter hormaechei* BD188	54 (20.15%)	20 (9.17%)	170.00%
*Kluyvera ascorbata* BD180	23 (8.58%)	17 (7.80%)	35.29%
*Providencia rettgeri* BD171	26 (9.70%)	17 (7.80%)	52.94%
*Citrobacter koseri* BD195	2 (0.75%)	11 (5.05%)	−81.82%
*Lactococcus garvieae* BD480	17 (6.34%)	5 (2.29%)	240.00%
*Providencia vermicola* BD232	15 (5.60%)	3 (1.38%)	400.00%
*Enterococcus faecium* BD402	11 (4.10%)	3 (1.38%)	266.67%
*Acinetobacter radioresistens* BD638	1 (0.37%)	2 (0.92%)	−50.00%
*Acinetobacter bereziniae* BD101	1 (0.37%)	0 (0.00%)	+
*Providencia alcalifaciens* BD401	0 (0.00%)	1 (0.46%)	−
*Morganella morganii* BD279	2 (0.75%)	0 (0.00%)	+
*Leclercia adecarboxylata* BD183	3 (1.12%)	0 (0.00%)	+
*Lactococcus lactis* BD202	9 (3.36%)	0 (0.00%)	+
*Pseudomonas abietaniphila* BD206	1 (0.37%)	0 (0.00%)	+
Sum of colonies	268	218	

*Notes*

The absolute and relative abundance of 17 bacterial strains from total 486 isolates were listed in the table. Percentage change (IR‐UN)/UN means the relative abundance of representative strains changes by irradiation.

IR: Irradiated samples; UN: un‐irradiated samples; +: The bacteria strain was only isolated in the irradiated sample; −: The bacteria strain was only isolated in the un‐irradiated sample.

### 3.2 Recovery of ecological fitness of sterile males by gut bacteria re‐infection

**Figure 7:**
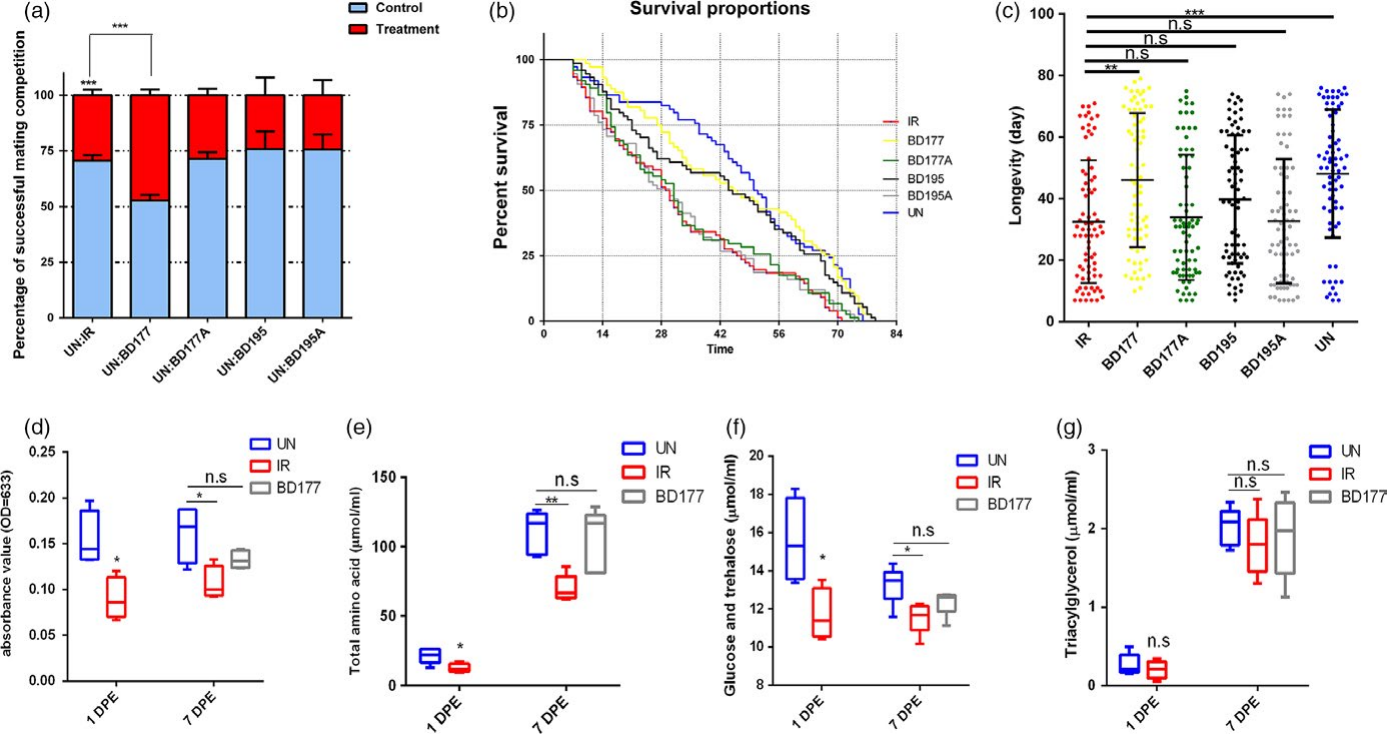
Commensal bacteria *K. oxytoca* BD177 resume the ecological fitness and improve the appetite and metabolism level in a sterile male insect. (a) The mating competition of irradiated male flies fed live *K. oxytoca* BD177 or *C. koseri* BD195 cells, autoclaved *K. oxytoca* BD177 or *C. koseri* BD195 cells, or conventional diet only, respectively, was measured, competed with un‐irradiated male flies which were fed conventional diet for the virgin female (*n* = 3~6). (b) Survival curves of different male populations by log‐rank analysis and Gehan–Breslow–Wilcoxon test. (c)The scatter diagram of adult longevity. (d) Food intake. Food consumption of male adult fly was measured by dye uptake at 1 DPE and 7 DPE (*n* = 5 experiments for each time point). (e~g) Sugar, lipid and amino acid levels. The 1DPE and 7DPE male fly (six male flies per each repetition, *n* = 4~6) were used. BD177: IR+ living I BD177 cells; BD177A: IR+ autoclaved *K. oxytoca* BD177; BD195: IR+ living *C. koseri* BD195 cells; BD195A: IR+ autoclaved *C. koseri* BD195 cells; IR: irradiated flies feeding on conventional diet only; UN: un‐irradiated male flies feeding on conventional diet only. The statistical tests were performed using *t* test between each different dietary group and control group. (*p < 0.05, **p < 0.001)

To confirm the role of key gut bacteria in the process of irradiation damage, flies were fed gut bacterial strains to assess whether gut bacteria re‐infection treatment could restore the ecological fitness damaged by ionizing radiation. Analysis of cultivable bacterial communities showed that the relatively abundant *C. koseri* and *K. oxytoca* were the top two descendants in the Enterobacteriaceae family reduced by irradiation, and *K. oxytoca* BD177 and *C. koseri* BD195 were identified as candidate bacteria. The prefix tests also revealed noticeable differences between UN and IR flies regarding fitness, that is, male fertility, flight capacity and survivorship (Table 1 and Figure 2). Flight capacity tests exhibited that flight duration, accumulative flight distance and the fastest flight speed in the IR flies fed with BD177 increased 1~2.5 times than that of IR flies fed without BD177 at 7 DPE (Table 3). A significant difference in the accumulative flight duration was observed consistently between IR flies fed with and without BD177 at 14 DPE (Table 4). Competitive mating experiments showed that IR males fed with BD177 accounted for 47% of the mating, which was not significantly different from the 53% mating rate observed in the UN control flies at 14 DPE (Figure 7a). The median lethal time was 30 days in IR male flies fed without BD177, which was significantly (*p* < 0.05) less than 45.5 days in male flies fed with BD177, whereas no difference was observed amongst the control flies regardless of feeding BD177 (Figure 7b). Adult longevity was significantly longer in IR male flies fed with BD177 than those fed without BD177, but no differences were observed amongst the control flies regardless of feeding BD177, as expected (Figure 7c). After feeding with alive BD195 bacteria, the survival rate of IR male flies was significantly enhanced (Figure 7b). On the other hand, autoclaved BD177 and the BD195 (including native and autoclaved bacterial cell) had no significant effect in improving the ecological fitness of IR male flies.

**Table 3:** Flight parameters of *B. dorsalis* adult males at 7 DPE

	Accumulative flight duration (h) (mean ± SE)	Accumulative flight distance (km) (mean ± SE)	Mean flight speed (m/s) (mean ± SE)	Fastest flight speed (m/s) (mean ± SE)
UN	0.90 ± 0.14**	1.64 ± 0.25**	1.83 ± 0.09	3.50 ± 0.12*
IR	0.42 ± 0.13	0.64 ± 0.18	1.62 ± 0.12	2.97 ± 0.18
BD177	0.90 ± 0.16**^,#^	1.62 ± 0.28**^,#^	1.73 ± 0.07	3.53 ± 0.10*^,#^
BD177A	0.43 ± 0.08	0.69 ± 0.13	1.59 ± 0.10	3.09 ± 0.23
BD195	0.28 ± 0.06	0.50 ± 0.12	1.63 ± 0.10	2.94 ± 0.17
BD195A	0.41 ± 0.13	0.67 ± 0.22	1.48 ± 0.10	2.91 ± 0.20

*Notes*

Tested at 26°C, no wind, and 60% RH for tethered‐flight 4 hr, *n* > 15. The statistical tests were performed using *t* test between each treatment group and control group.

BD177: IR+ live *K. oxytoca* BD177 cells; BD177A: IR+ autoclaved *K. oxytoca* BD177; BD195: IR+ live *C. koseri* BD195 cells; BD195A: IR+ autoclaved *C. koseri* BD195 cells; IR: irradiated flies feeding on conventional diet only; UN: un‐irradiated male flies feeding on conventional diet only.

*p < 0.05 versus IR, **p < 0.05 versus IR, ^#^ *p* < 0.05: BD177 versus UN.

**Table 4:** Flight parameters of *B. dorsalis* adult males at 14 DPE

	Accumulative flight duration (h) (mean ± SE)	Accumulative flight distance (km) (mean ± SE)	Mean flight speed (m/s) (mean ± SE)	Fastest flight speed (m/s) (mean ± SE)
UN	0.96 ± 0.19*	1.81 ± 0.34*	1.84 ± 0.11	3.77 ± 0.18
IR	0.36 ± 0.08	0.84 ± 0.24	1.60 ± 0.15	3.95 ± 0.64
BD177	0.92 ± 0.23*^,^#	1.75 ± 0.49*^,^#	1.84 ± 0.11	4.07 ± 0.38
BD177A	0.71 ± 0.14	1.17 ± 0.24	1.68 ± 0.11	4.28 ± 0.37
BD195	0.52 ± 0.16	0.84 ± 0.25	1.60 ± 0.14	3.84 ± 0.50
BD195A	0.59 ± 0.15	1.03 ± 0.25	1.75 ± 0.16	4.43 ± 0.54

Tested at 26°C, no wind, and 60% RH for tethered‐flight 4 h, *n* > 15. UN: un‐irradiated male flies feeding on conventional diet only; IR: irradiated flies feeding on conventional diet only; BD177: IR+ live K. oxytoca BD177 cells; BD177A: IR+ autoclaved K. oxytoca BD177; BD195: IR+ live C. koseri BD195 cells; BD195A: IR+ autoclaved C. koseri BD195 cells. The statistical tests were performed using *t*‐test between each treatment group and control group. *p < 0.05 vs IR, #*p* < 0.0.05: BD177 vs UN.

### 3.3 Improved food intake and haemolymph nutrient level in sterile males re‐infected with gut bacteria

To further explore the physiological mechanism for restoring ecological fitness, firstly, we investigated the influence of irradiation on food intake and major nutrient levels, that is, sugars, amino acids and lipids, in the haemolymph of *B. dorsalis*. The results showed that irradiation significantly affected food intake and major nutrient levels, that is, sugars and amino acids (Figure 7d–f), but did not affect lipid levels in the haemolymph of flies (*p* > 0.05) (Figure 7g). At 1 DPE, food intake (*p* = 0.0141), total sugars (*p* = 0.0247) and total free amino acids (*p* = 0.0144) of IR male flies were significantly lower than their counterparts in the control (Figure 7d–f). The food intake and major nutrient (sugar and amino acid) levels in the haemolymph of IR male flies were also consistently and significantly lower (*p*‐values of 0.0244, 0.0181 and 0.0010, respectively) than those in controls at 7 DPE (Figure 7d–f).

Secondly, we investigated whether food intake and major nutrient, that is, sugar, lipid and amino acid levels in the haemolymph of IR male flies could be recovered via feeding intestinal probiotics. Intriguingly, we found that the food intake and total free amino acid of BD177‐ re‐infected IR flies was significantly higher (*p*‐values of 0.0475 and 0.0124, respectively) than the counterparts of IR male flies without feeding BD177, whereas no significant difference (*p* > 0.05) was observed in the food intake and major nutrient, that is, sugar, lipid and amino acid levels between IR flies fed with BD177 and the UN flies (Figure 7d–f). These results indicated that food intake, and sugar and amino acid levels in the haemolymph of IR male flies were recovered by the supplementation of BD177 probiotic.

## 4 DISCUSSION

Sterile insect technique is an environmentally friendly control method that was previously applied to B. dorsalis (Orankanok et al., 2007). However, a crucial issue for the successful and practical implementation of SIT on a large scale is how to produce a large number of high quality sterile males, that can compete with wild males for mating. The ecological fitness of sterile males should be considered before any SIT application. It has been shown earlier that gut symbiotic bacteria play a prominent role in promoting ecological fitness of their insect hosts (Engel & Moran, 2013). Thus, we investigated the possibility of enhancing ecological fitness by manipulation of the gut microbiota. We first demonstrate that irradiation damage on host ecological fitness was interlinked with intestinal microbial community dysbiosis, using the classic sterile male insect model. Next, we show that supplementation of gut bacteria to sterile male *B. dorsalis* reversed their reduced ecological fitness level via enhancement of nutrient uptake.

The potential relationship between intestinal bacterial communities and ecological fitness damage of *B. dorsalis* was observed in first. We found that ionizing radiation resulted in a decline in male mating ability, flight capacity and survivorship, in agreement with previous studies (Helinski et al., 2009; Lance & McInnis, 2005; López‐Martínez & Hahn, 2014). In our study, the assessment method of mating competitiveness and life span parameter of sterile males was as in previous reports (Collins et al., 2009). But the parameters of flight ability were evaluated by flight mills equipment different from the conventional method of SIT. Flight mills are commonly used for the study of insect flight and provide detailed parameters such as speed and distance of flight (Attisano et al., 2015). We think that flight mills can be applied in the quality evaluation of sterile males.

Then the analysis of gut microbiota community showed that irradiation had a remarkable shift in the bacterial composition and structure, as well as a characteristic of higher gut microbial richness and diversity. In addition, both the relative abundance and the load of the intestinal bacterial community such as Enterobacteriaceae significantly decreased, whereas the minor members of the gut community (Bacillaceae, Clostridiaceae, Xanthomonadaceae, Sphingobacteriaceae, Aeromonadaceae and Flavobacteriaceae) increased significantly. Although the perturbation of gut microbiota by general external factors has been well studied (David et al., 2014; Perez‐Cobas et al., 2013), little is known about how ionizing radiation influences the intestinal microbiota in metazoa. In humans, intestinal microflora diversity, richness and the Firmicutes/Bacteroidetes ratio were significantly altered by radiotherapy in pelvic radiotherapy patients who later developed diarrhoea (Wang et al., 2015). Similarly, bowel irradiation may lead to a general decrease in gut microbiota, an imbalance of the gut bacterial community structure and subsequent pathogenic effects on the epithelial mucosa (Johnson et al., 2004). In mice, localized internal rectal radiation resulted in a reduced diversity of the Firmicutes and Bacteroidetes and increased in Proteobacteria (Gerassy‐Vainberg et al., 2018). In addition, gut microbes have been shown to regulate intestinal radiosensitivity of mice ((Crawford & Gordon, 2005). In insects, γ‐ray radiation was also found to reduce the major members of the gut bacteria community of the Mediterranean fruit fly (Ben Ami et al., 2010). Few examples from above studies showed that ionization radiation can affect not only fitness of host but also its gut microbiota.

The gut microbial homoeostasis often contributes to the fitness of the host, and perturbing it can have harmful effects on the host. Disturbance of gut microbial homeostasis makes the host more susceptible to pathogens (Blumberg & Powrie, 2012), impairing intestinal epithelium (Ryu et al., 2008) and functions (Clark et al., 2015), as well as resulting in increased mortality (Clark et al., 2015; Li, Qi, & Jasper, 2016; Raymann, Shaffer, & Moran, 2017). Dramatic changes of microbiome taxonomy tend to drive microbiome functional shifts (Manor & Borenstein, 2017). Our PICRUSt analysis results showed that the increased relative abundance of the minor gut community members positively correlates with an increase in amino acid, carbohydrate, lipid, cofactor and vitamin metabolism in IR male flies. The high relative abundance of minor members of the gut bacterial community, including Bacillaceae, Xanthomonadaceae and Aeromonadaceae, tended to be harmful to the host as previously reported. For example, *Bacillus cereus* could stimulate the immune response of the adult *B. dorsalis* by inducing the expression of immune‐related genes (Duox) and increased ROS levels at 6 hr post oral infection (Yao et al., 2016). Members of the Xanthomonadaceae can use chitin as a carbon source (Killiny, Prado, & Almeida, 2010) and results in its degradation, which (chitin) is a major structural compound in insect guts (Engel & Moran, 2013). Therefore, a high relative abundance of Xanthomonadaceae may destroy the structure of the gut. In the same way, numerous members of Aeromonadaceae can cause infections in insects and crustaceans by the lipopolysaccharides (Noonin et al., 2010). Recent studies have suggested that perturbations of the gut microbiota may foster “blooms” of otherwise low‐abundance and harmful bacteria, which often directly compete with the host for metabolic substrates, such as carbon sources and amino acids (Olive & Sassetti, 2016). Once dysbiosis is established, potential pathogens can rapidly outcompete commensals due to factors in their genomes that confer greater resistance to host defence mechanisms (e.g., antimicrobial peptides and reactive oxygen species) and better utilization of the gut nutrient environment (Pham & Lawley, 2014). Taken together, increased minor members of the gut community would contribute to a distinct metabolic milieu in the digestive tract including elevated metabolic levels of primary nutrient metabolites (carbohydrates, lipids and amino acids, etc.) and elevated host immune response that could in turn negatively impact host fitness.

The prominent gut microflora of insects often plays an important role in the maintenance of nutrition, development and ecological behaviour of insects (Engel & Moran, 2013). In our previous study, we have revealed Enterobacteriaceae as a stable and dominant intestinal commensal bacterial community in the gut of *B. dorsalis* (Wang, Jin, et al., 2014; Wang et al., 2011) and as a source of metabolites which attract *B. dorsalis* adults (Shi et al., 2012). In this study, irradiation caused a significant reduction in the relative abundance and the load of the fundamental microflora Enterobacteriaceae in *B. dorsalis* sterile male. Recently, a study showed that Enterobacteriaceae can degrade trichlorphon and conferred host insecticide resistance in *B. dorsalis* (Cheng et al., 2017). In other insects, these bacteria may contribute to nitrogen fixation (Behar et al., 2005), they may affect mating latency (Ben Ami et al., 2010) and pheromone synthesis (Wada‐Katsumata et al., 2015) and they may play essential role to host fitness by preventing the establishment or proliferation of pathogenic microorganism (Cirimotich et al., 2011; Koch & Schmid‐Hempel, 2011). Thus, the reduction of the Enterobacteriaceae main flora in *B. dorsalis* gut may result in decreased nitrogen fixation, blocked pheromone synthesis and flourishing in minor members of the gut microbiota, which then lead to various features of ecological fitness decline in sterile males.

To confirm the role of key gut bacteria in irradiation damage process, an analysis of the culturable bacteria profile was first carried out. According to gut bacteria re‐infection experiments, we found that a bacteria strain *K. oxytoca* BD177 belonging to Enterobacteriaceae played a key role in restoring host ecological fitness damaged by irradiation. Our results showed that by feeding *K. oxytoca* BD177 to IR sterile males, ecological fitness including male mating competitiveness, some parameters of flight capacity, survival rate and life span of IR males group could be restored to the level of UN males group, which suggests that irradiation influence host ecological fitness via dysbiosis of intestinal microbial community. Our results are in accordance with the results of some previous studies. For instance, in *D. melanogaster*,* Lactobacillus plantarum* is responsible for mating preference and the composition of the gut microbiota determines mating attractiveness: flies mated preferentially with individuals harbouring similar microbiota, and this preference was probably linked to microbiota‐dependent variation in the cuticle hydrocarbon profile (Sharon et al., 2010). Similarly, in *Libellula pulchella*, protozoan parasite infections that affect gut function can alter flight muscle development and decrease flight performance (Schilder & Marden, 2007). A study of *C. capitata* showed that feeding gut commensal Enterobacteriaceae bacteria extended host longevity (Behar et al., 2008). However, in our study, *K. oxytoca* BD177 supplement to sterile males showed that the ecological fitness of IR flies has been restored for some but not for all measured parameters. One explanation for the recovery of this could be that irradiation can damage the midgut tissue of sterile insect males (Lauzon & Potter, 2012) so that *K. oxytoca* BD177 may not persistently colonize in the damaged midgut for playing a beneficial role on host fitness.

When sterile males were supplemented with the *K. oxytoca* BD177, the food intake and major nutrients level of IR male flies were recovered (Figure 6d–g). As reported by previous studies of vertebrate, gut bacteria might play an important role in the control of host appetite. Breton and his colleagues found that administration of proteins ClpB of gut bacteria *Escherichia coli* belonging to Enterobacteriaceae affected host food intake, depending on *E. coli* growth phases (Breton et al., 2016). Enterobacteriaceae components such as lipopolysaccharide or short‐chain fatty acids of bacterial metabolism were reported in relation to appetite control and energy balance (Fetissov, 2016). In the invertebrate study, the dominant bacteria of *D. melanogaster* (Acetobacter and Lactobacillus) significantly affect food preferences and foraging behaviour of its host by modulating olfaction (Wong et al., 2017). We also found in a previous study that metabolites from *K. oxytoca* BD177 can attract *B. dorsalis* adults (Shi et al., 2012). Thus, it is possible that *K. oxytoca* BD177 regulates the feeding behaviour of irradiated males by attracting them and inducing them to ingest more food. In addition, *K. oxytoca* have the capacity for nitrogen fixation and the conversion of N_2_ to ammonia (Temme, Zhao, & Voigt, 2012). It is known that nitrogen fixation can influence plant growth (Pavlova et al., 2017) and insect longevity (Behar et al. 2008). Another strain of *K. oxytoca* isolated from the gut of *Poekilocerus pictus* was shown to degrade oleic acid to produce the smaller‐chain fatty acids (Kingsly, Jothinathan, & Thilagaraj, 2017). This bacteria species has also been found to utilize glycerol and xylose as the carbon source to yield chemical products (Cho et al., 2015). These studies suggested that *K. oxytoca* has the ability to exploit the nitrogen and carbon sources of the environment. Thus, *K. oxytoca* BD177 may also contribute to the utilization of amino acids and sugars from the diet.

Several studies have considered the effect of adding probiotics to the diet of Tephritidae fruit flies, especially in medfly in order to enhance the efficiency of SIT. Ben Ami and his colleagues reported that the supplementing *Klebsiella* spp. to adult irradiated medflies significantly shortened the mating latency of the sterile male *C. capitata* (Ben Ami et al., 2010). A larger size and better mating competitiveness of males have also been observed by administering a cocktail of live bacteria (*K. pneumonia*,* Enterobacter* spp. and *C. freundii*) into the larval diet of *C. capitata*. Provision of the *Enterobacter* sp. AA26 strain resulted in bigger pupal size and shorter developmental duration, as well as decreased mortality during immature stages of *C. capitata* (Augustinos et al., 2015).

Overall, our study showed that irradiation induced dysbiosis of the gut bacterial community with an elevated diversity where major members such as Enterobacteriaceae decreased and minor members increased, leads to higher nutrient‐related metabolism activity of gut bacteria in irradiated male flies due to the increased minor bacteria community. Moreover, we also observed that food intake of IR flies declined. Therefore, both lower intake of food and higher nutrient consumption of flourishing minor gut microbiota would cause host nutrients and energy metabolic activity decline in IR flies, which then resulted in the downfall in the ecological fitness of IR flies. Those results suggest a link between gut community dysbiosis and host ecological fitness. Our results may enlighten similar side effects caused by irradiation injury in cancer radiotherapy where patients often exhibit general symptoms such as anorexia, diarrhoea, malnutrition and intestinal inflammation after radiation therapy (Touchefeu et al., 2014). Therefore, we suggest further efforts to investigate if the administration of intestinal probiotics can also improve the health status of patients undergoing radiotherapy. However, in the current study, we highlighted the possibility of insect‐gut associated microbiota (probiotics), for example live *K. oxytoca* could be the unexplored but encouraging source to exploit for repairing the irradiation damage and producing higher quality sterile insects, with equal fitness levels to wild males for pest control strategies and enhanced efficiency of SIT applications.

## CONFLICT OF INTEREST

None declared.

## AUTHORS’ CONTRIBUTIONS

H.Y.Z. conceived and designed the project. Z.H.C. designed and performed experiments and analysed all data. Z.C.Y designed experiments and analysed the data of next‐generation sequencing. Y.S.L, Z.Z, S.B performed fitness assay, isolation and identification of microorganisms. H.Y.Z., Z.H.C., Z.C.Y., Z.X. and K.B. wrote the manuscript. All authors read and approved the final manuscript.

## DATA ACCESSIBILITY

The entire 16S *rRNA* gene sequence dataset can be retrieved from the National Center for Biotechnology Information Sequence Read Archive (accession no. PRJNA413124).
