# Supplementary material for: Intestinal probiotics restore the ecological fitness decline of Bactrocera dorsalis by irradiation
Source: Evol Appl. 2018 Oct 9;11(10):1946–63. doi: 10.1111/eva.12698 (PMC6231467; doi:10.1111/eva.12698)

**Figure S1** Male fertility, the egg hatching. UN: un-irradiated male flies ; IR: irradiated male flies. Data were analyzed using Student’s test. The error bars indicate standard error (SE). (* P$<$0.05, ** P$<$0.01,*** P$<$0.001).


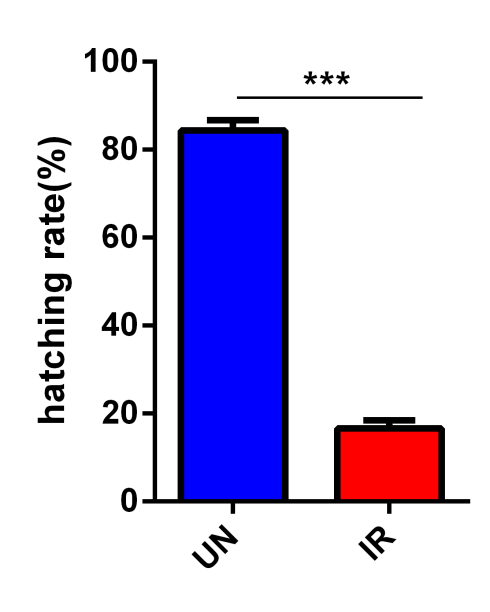

Supplement: Supplementary file 1 [file EVA-11-1946-s001.docx]
