# Supplementary material for: Intestinal probiotics restore the ecological fitness decline of Bactrocera dorsalis by irradiation
Source: Evol Appl. 2018 Oct 9;11(10):1946–63. doi: 10.1111/eva.12698 (PMC6231467; doi:10.1111/eva.12698)
Supplement: Supplementary file 2 [file EVA-11-1946-s002.docx]

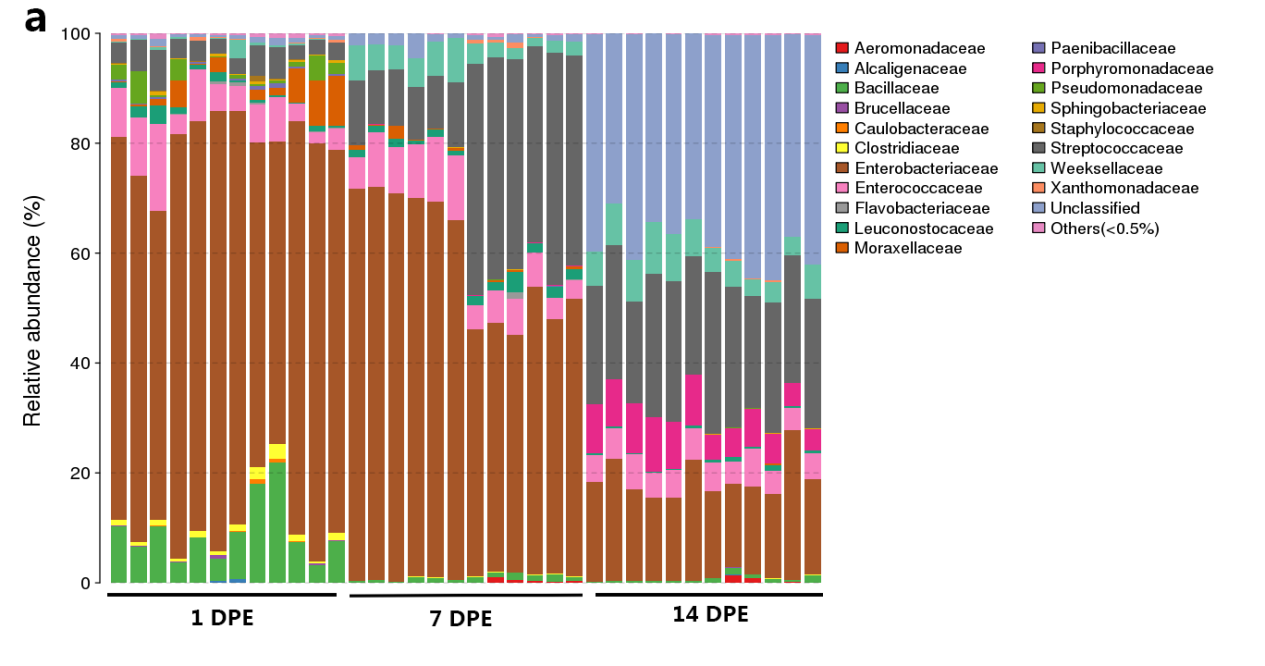
**­­**

**Figure S2** Quantitative histogram of differential features from family level percent relative abundance data. (a)Taxonomic breakdown at the family level grouped by un-irradiated and irradiation samples. (b–j) Relative abundance of different bacterial taxa in un-irradiated and irradiated samples at 1, 7 and 14 DPE. Statistical comparison was based on Student’s t-test (*P＜0.05，**P＜0.01，***P＜0.001). The error bars indicate standard error (SE).UN: un-irradiated male fly; IR: irradiated male fly.
