# Supplementary material for: Intestinal probiotics restore the ecological fitness decline of Bactrocera dorsalis by irradiation
Source: Evol Appl. 2018 Oct 9;11(10):1946–63. doi: 10.1111/eva.12698 (PMC6231467; doi:10.1111/eva.12698)
Supplement: Supplementary file 3 [file EVA-11-1946-s003.docx]

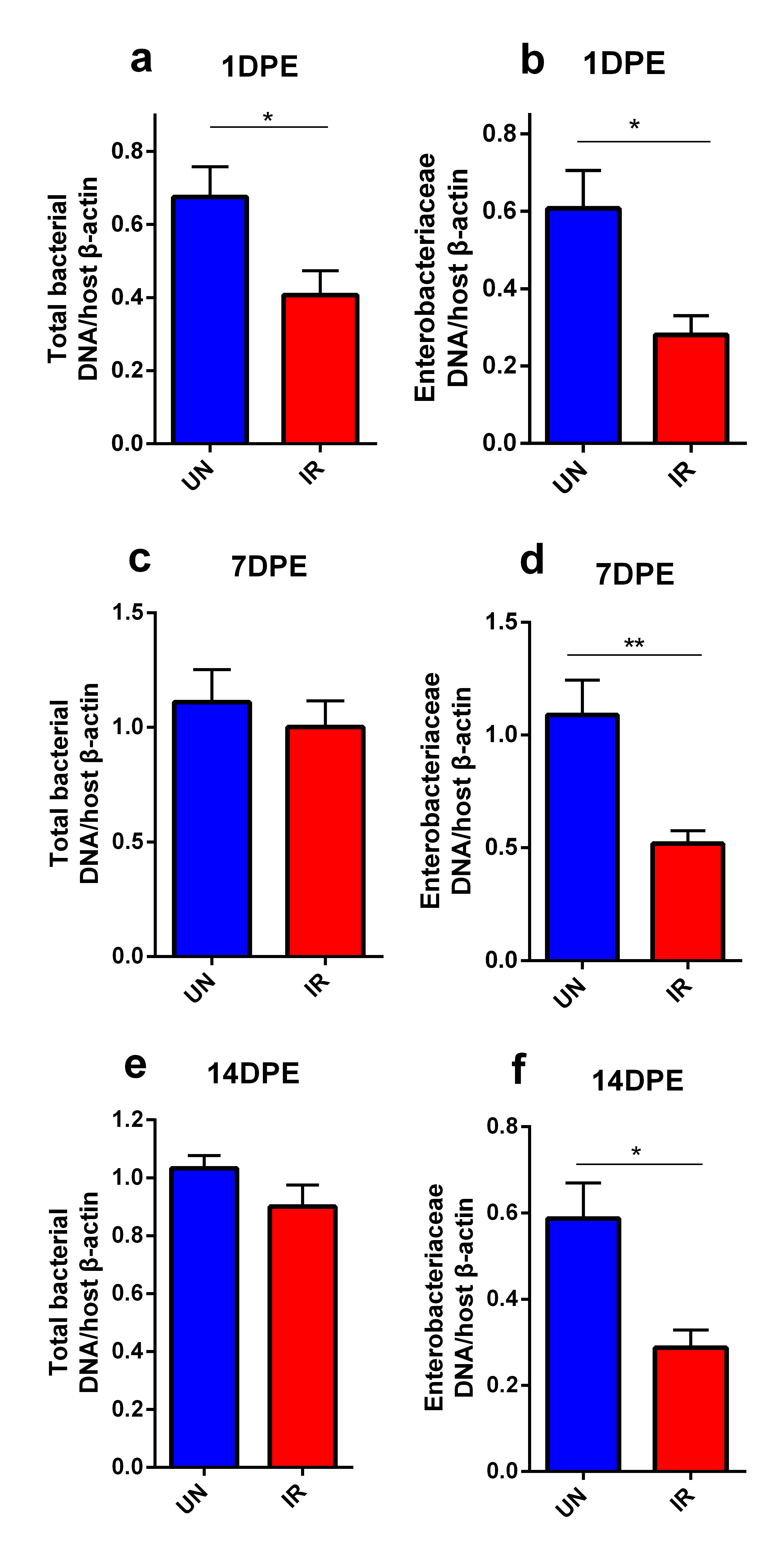


**Figure S3** Total bacteria and Enterobacteriaceae were quantified by realtime PCR using primers for species-specific 16S rRNA genes. Data were normalized by real-time PCR data for the β-actin gene in host genomic DNA. Data were analyzed using Student’s test. The error bars indicate standard error (SE). (* P$<$0.05, ** P$<$0.01,*** P$<$0.001).
