# Supplementary material for: Intestinal probiotics restore the ecological fitness decline of Bactrocera dorsalis by irradiation
Source: Evol Appl. 2018 Oct 9;11(10):1946–63. doi: 10.1111/eva.12698 (PMC6231467; doi:10.1111/eva.12698)

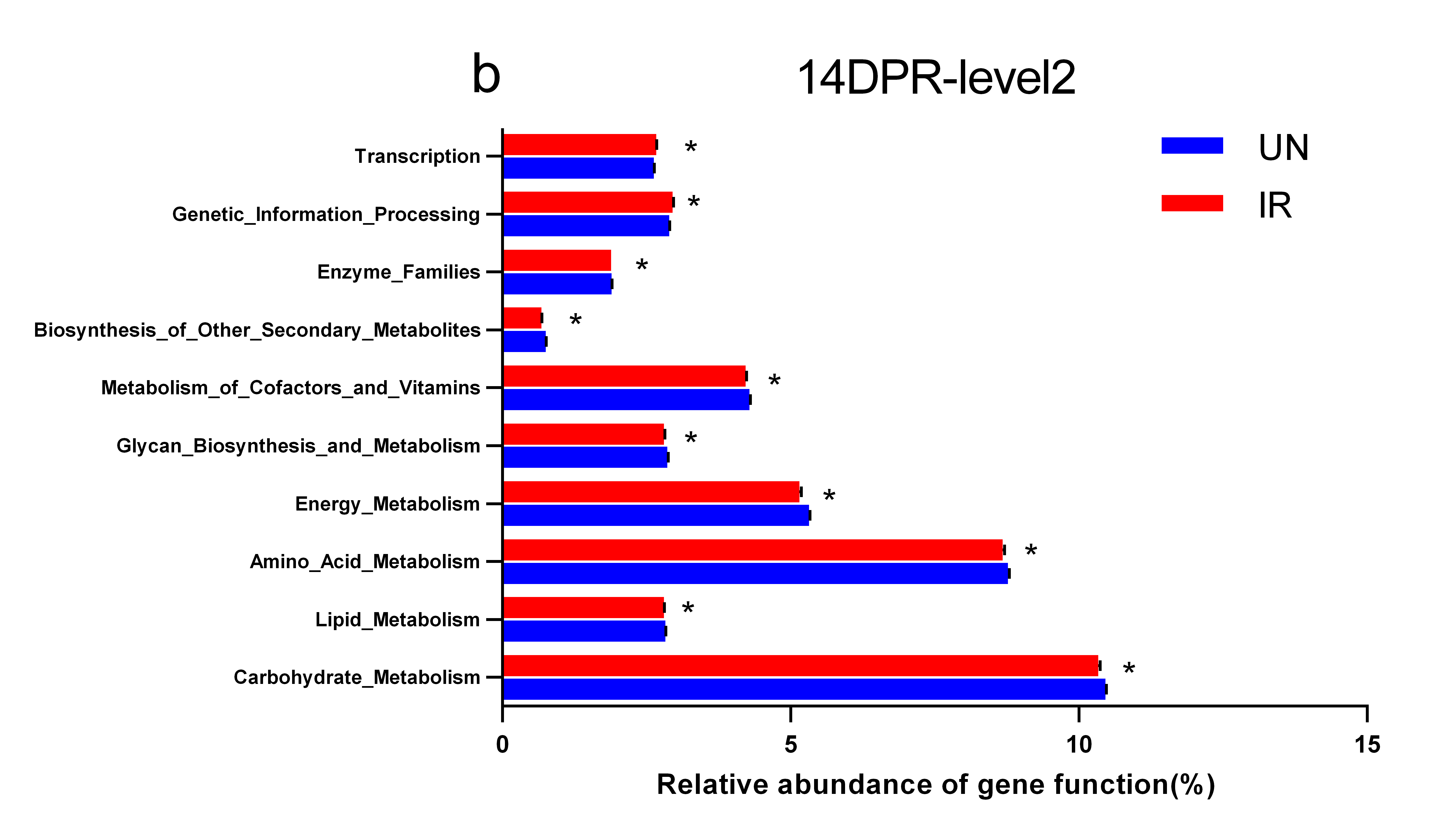

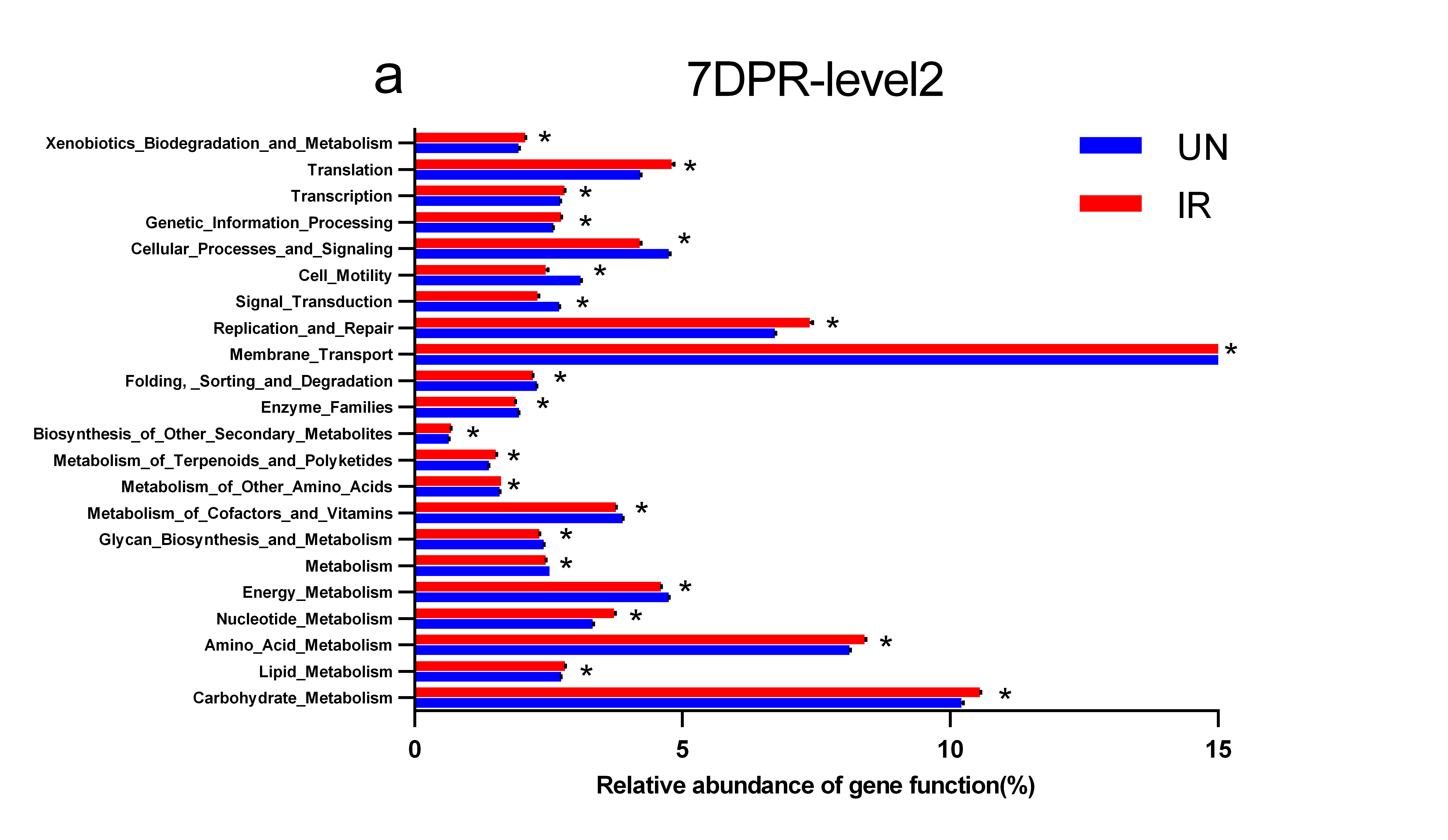


**Figure S4** Bacterial metabolic and other pathway diﬀerences in the gut samples of UN *vs.* IR male fly. (a~b) Comparison in the relative abundance of PICRUSt-generated functional profile of gut microbiota and significant differences in gene categories at level 2 (t-test, P< 0.05) between the irradiated group and the control group. The function of gut microbiome of un-irradiated (UN) and irradiated (IR) male flies at 7 DPE (a, n=6) and 14 DPE (b, n=6). The error bars indicate standard error (SE). (* P$<$0.05).


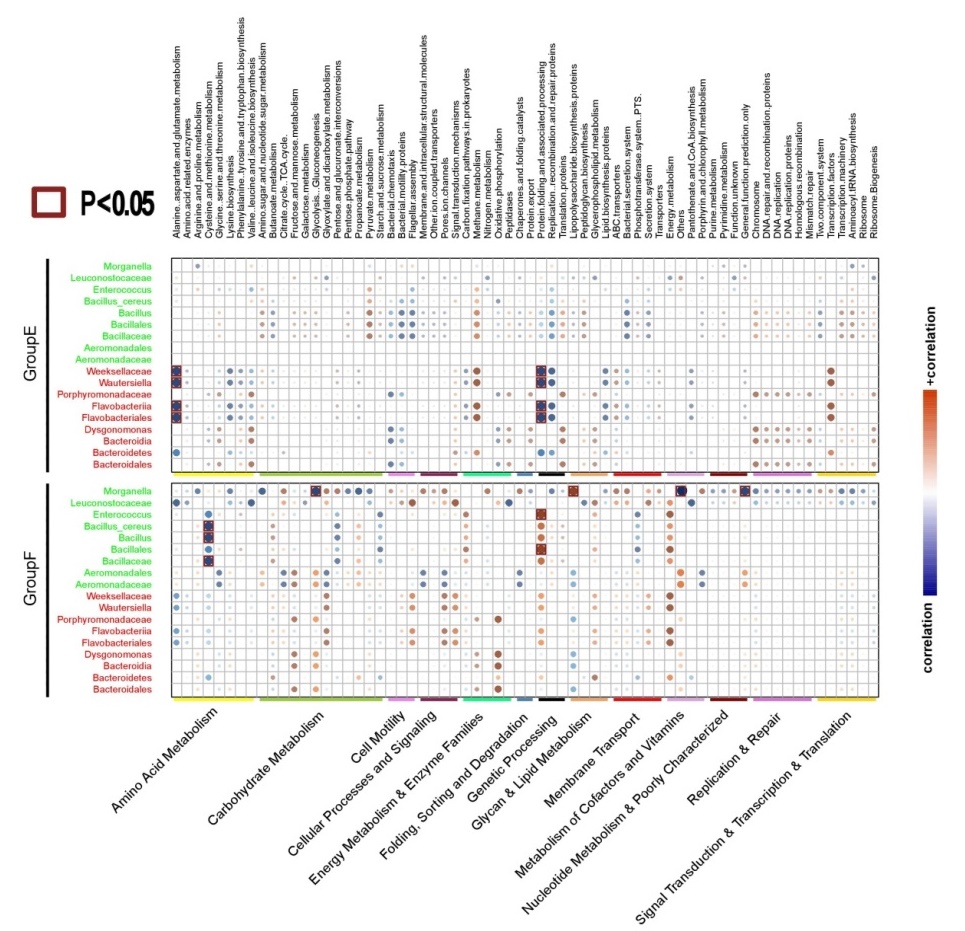

Supplement: Supplementary file 4 [file EVA-11-1946-s004.docx]
