# Supplementary material for: Intestinal probiotics restore the ecological fitness decline of Bactrocera dorsalis by irradiation
Source: Evol Appl. 2018 Oct 9;11(10):1946–63. doi: 10.1111/eva.12698 (PMC6231467; doi:10.1111/eva.12698)
Supplement: Supplementary file 5 [file EVA-11-1946-s005.docx]

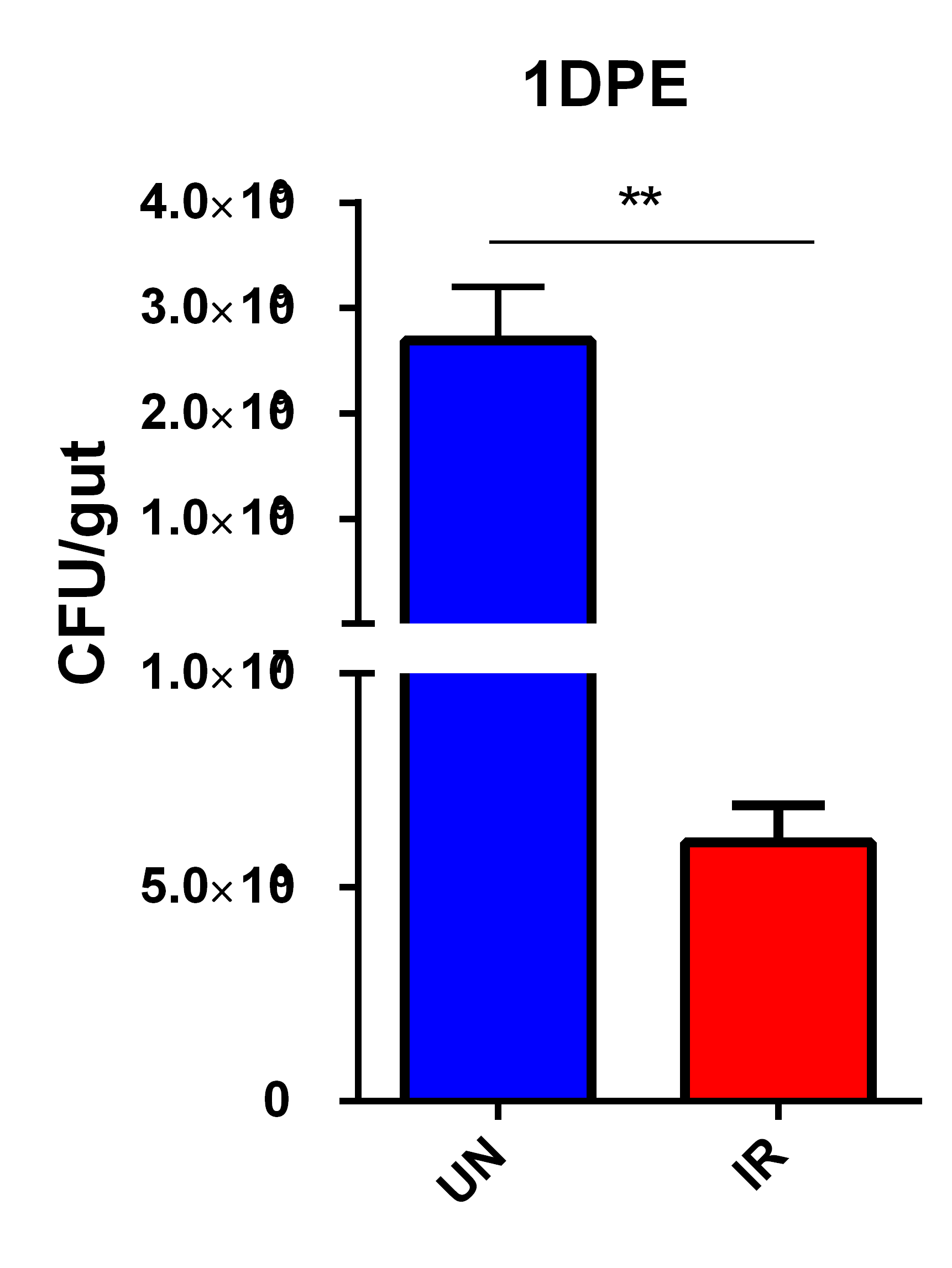

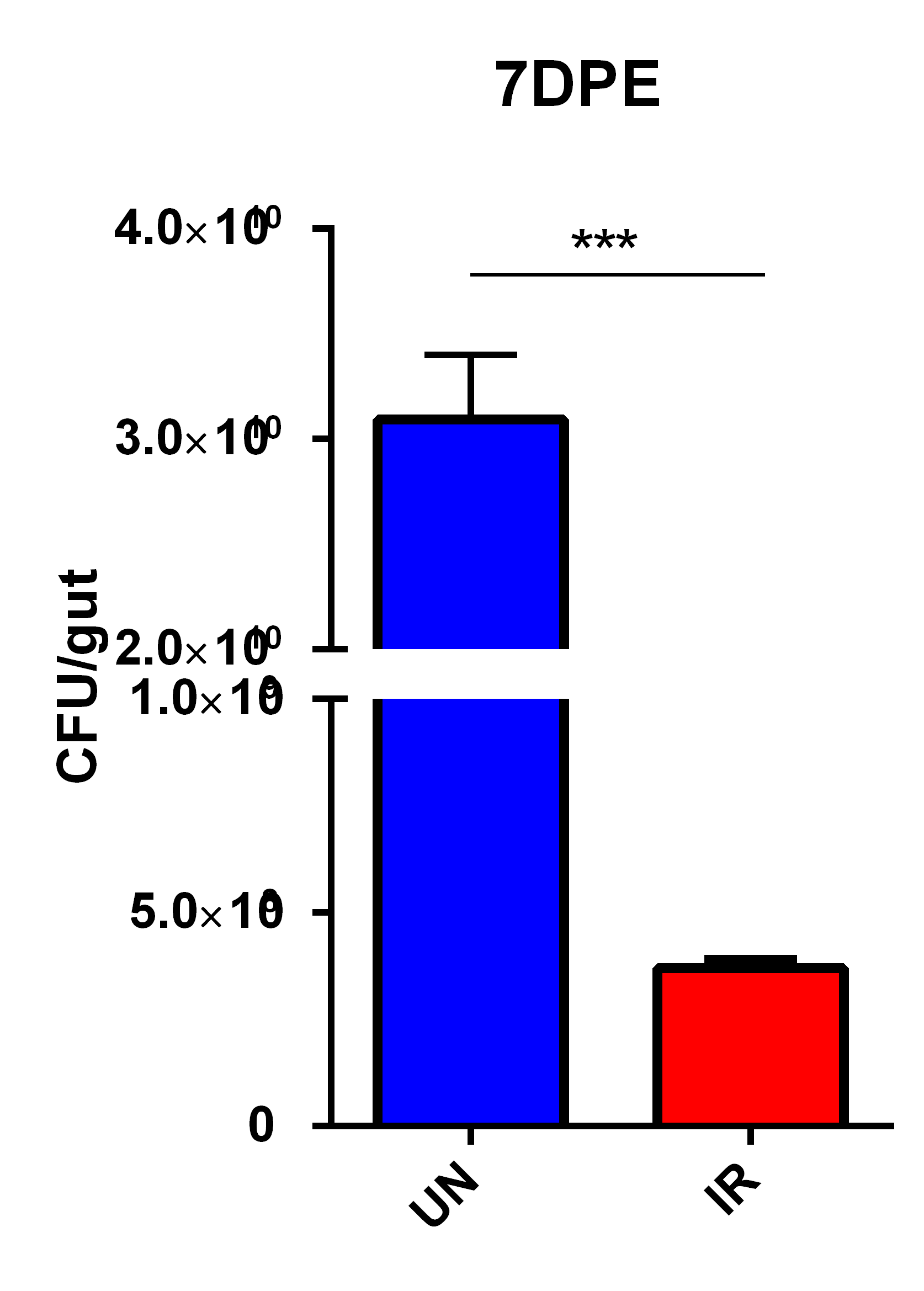

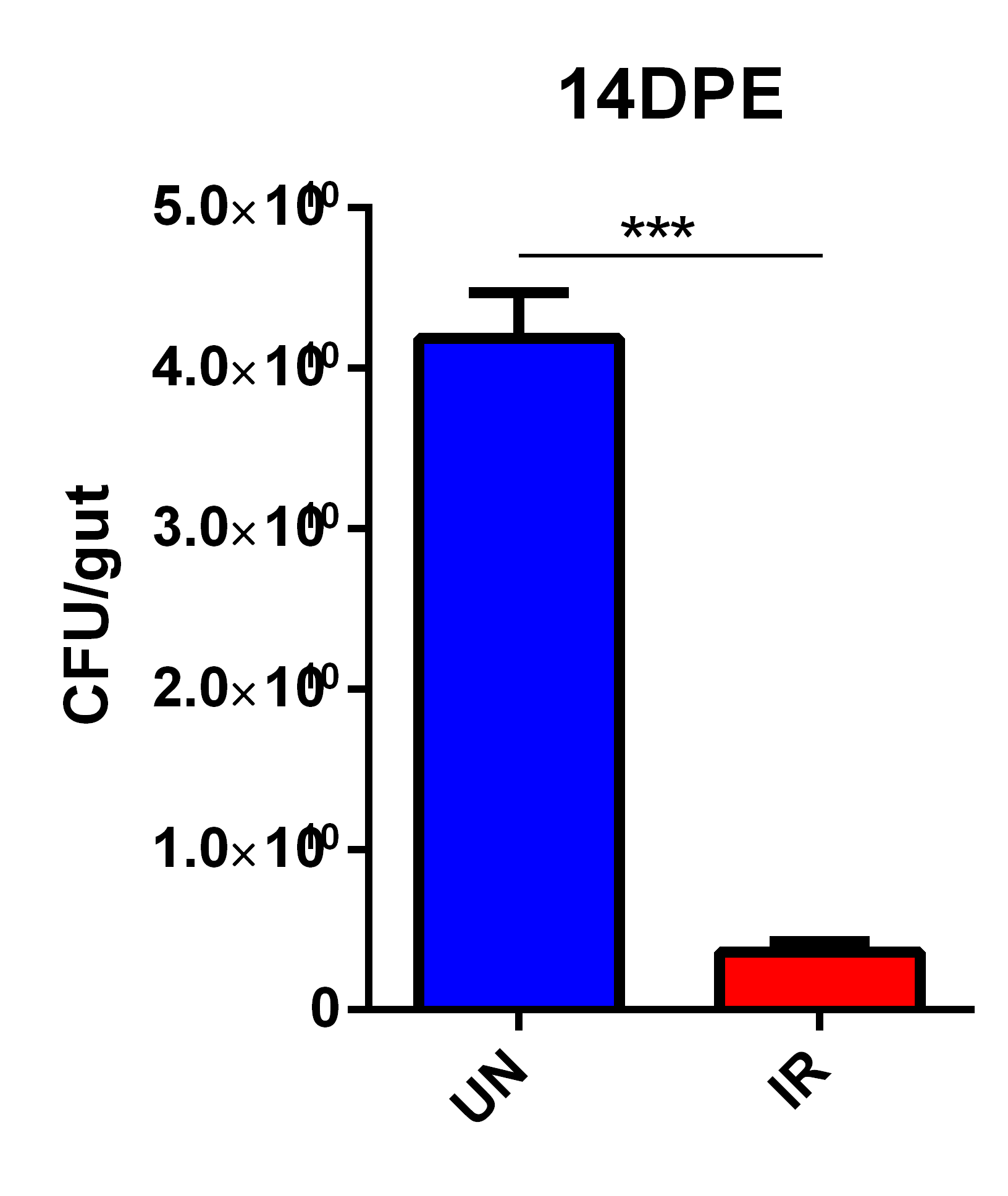


a

b

c

**Figure S5** Radiation reduced the cultivable bacteria load of male flies. (a~c) colony-forming unit (CFU) of the irradiated and un-irradiated male flies at 1 DPE, 7 DPE and 14 DPE (n=3). Data were analyzed using Student’s test. The error bars indicate standard error (SE). (* P$<$0.05, ** P$<$0.01,*** P$<$0.001).
