# Supplementary material for: Intestinal probiotics restore the ecological fitness decline of Bactrocera dorsalis by irradiation
Source: Evol Appl. 2018 Oct 9;11(10):1946–63. doi: 10.1111/eva.12698 (PMC6231467; doi:10.1111/eva.12698)
Supplement: Supplementary file 6 [file EVA-11-1946-s006.docx]

**Figure S6** Phylogenetic relationships of 486 cultivable gut bacteria strains. This maximum-likelihood built with IQ-TREE is based on 16s rRNA gene aligned by indicating the relationship between 486 gut bacteria strains with 63 reference bacteria from NCBI database. The tree is inferred under the TIM3+F+R4 model with ML ultrafast bootstrap support (-bb 1000).


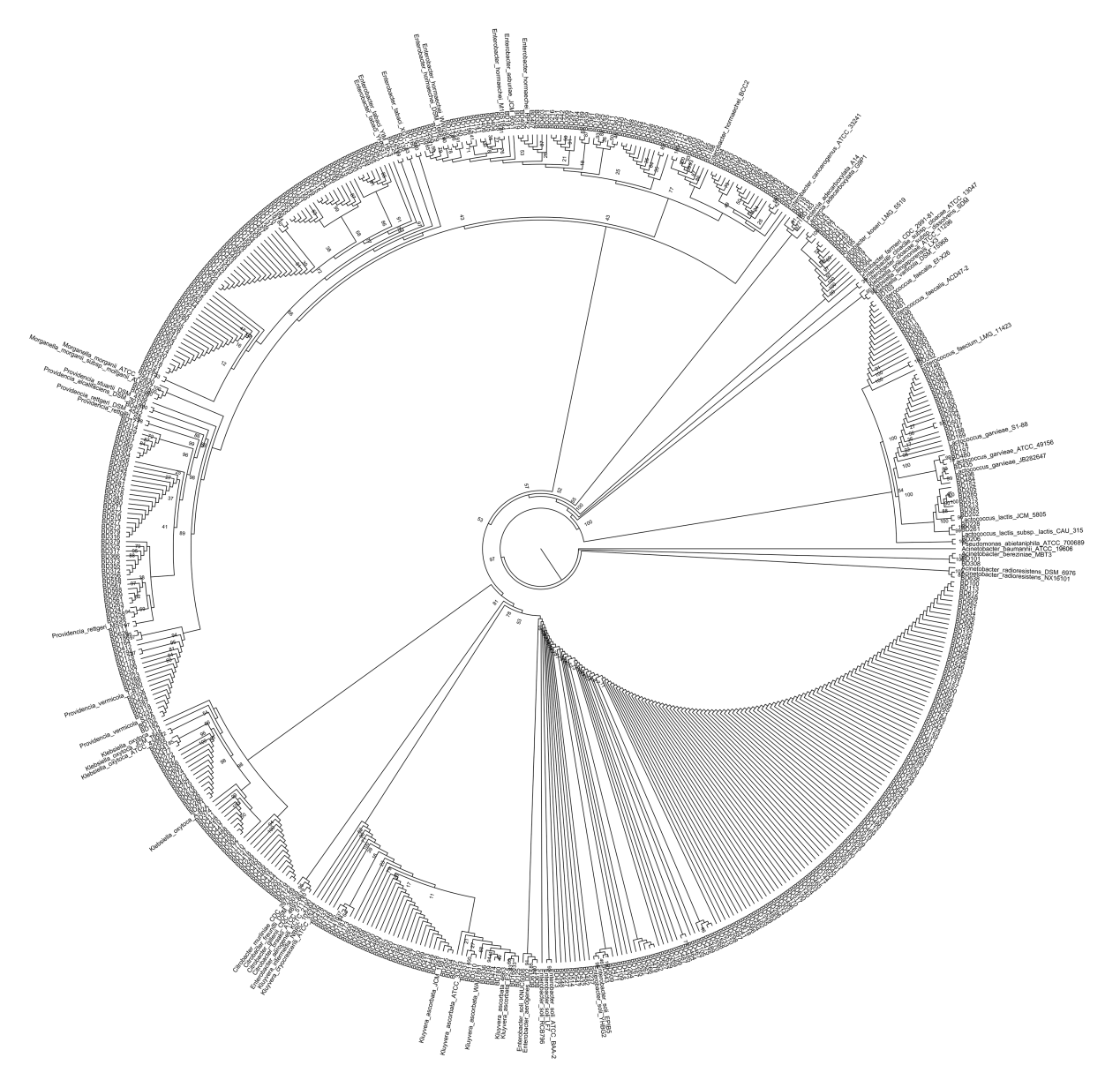


­

**Figure S6** Phylogenetic relationships of 20 representative cultivable gut bacteria strains. Phylogenetic tree based on16sRNA gene sequences aligned by indicating the relationship between these bacteria with type strains, using neighbor-joining method (1 000 bootstrap replicates) in MEGA version 7.
